# Research on the sustainability of "greening" process in the Mu Us Sandy Land based on the spatiotemporal stability of ecological land

**DOI:** 10.1371/journal.pone.0292469

**Published:** 2024-02-06

**Authors:** Qiumeng Zhang, Baoquan Jia, Tong Li, Wenrui Li

**Affiliations:** 1 Research Institute of Forestry, Chinese Academy of Forestry, Beijing, China; 2 Key Laboratory of Tree Breeding and Cultivation, National Forestry and Grassland Administration, Beijing, China; 3 Urban Forest Research Center, National Forestry and Grassland Administration, Beijing, China; Ningbo University, CHINA

## Abstract

In environmentally sensitive areas, especially the arid and semi-arid regions, the greening stability process and its influencing factors can directly affect the sustainable development of the ecological environment. In this study, multi-source remote sensing data such as land use/cover data, MODIS NDVI, and soil moisture, methods such as stability index, vegetation quantitative remote sensing, and Geodetector were employed to analyze the sustainability of the greening process in the Mu Us Sandy in 2000–2020, which were viewed from three aspects: changes in stability of land use types and function, soil moisture change and influencing factors on greening stability. The results showed that, (1) From the stability of land use types, continuous stable ecological land accounted for more than 50%, showing that decreased from northwest toward southeast. (2) From the functional stability, NDVI showed a fluctuated growth (0.035/a), with an increasing distribution pattern from northwest to southeast. Additionally, Vegetation changes were unstable and concentrated in the western part of the study area (OtogBanner and Otog Front Banner), while the eastern part was stable, in which vegetation improvement took the main position. Moreover, mobile dunes almost disappeared, and semi-fixed dunes decreased and gradually shrank to the west of the sandy area, while fixed dunes soared and were concentrated in the middle of the sandy land. (3) From the soil moisture change, soil moisture at different underground depths showed an overall increasing trend, but the deep soil moisture was higher than the shallow, and spatial distribution varied greatly. (4) From the influencing factors, natural factors significantly influence greening stability, among which precipitation had a particularly profound impact, and interactions with other natural and social factors were higher explanatory. The paper aims to explore whether the ecological environment is developing in a good and orderly direction in the Mu Us Sandy Land, and the potential factors that cause its changes, to provide a theoretical basis for scientific governance in the Mu Us Sandy Land and other arid and semi-arid areas in the future.

## Introduction

Since the 1980s, in the face of the increasing ecosystem degradation under the influence of global warming and human activities, the international scientific community has initiated and organized major scientific research on global change and earth system. Indeed, ecological construction activities centered on vegetation restoration have been implemented in various countries around the world, and remarkable progress has been made, as evidenced by global greening trends based on various data sources [1–3]. In terms of countries, China has the highest contribution to global greening, reaching 25% [1]. Northern China, located in the agro-pastoral zone and the northern edge of the East Asian monsoon, has seen significant greening in the past two decades due to ecological restoration projects and grazing prohibition policies [4–6], concerns have been raised by scholars about the long-term stability of the concentrated greening due to the unique geographical location and fragile natural environment of this region [7, 8].

Terrestrial vegetation is the primary producer of ecosystems and an important component of terrestrial ecosystems [9]. It plays an important role in natural ecological services such as climate regulation, carbon cycle, and energy exchange on different time scales from regional to global scales [10, 11]. Among them, ecological land, including forest and grassland with ecological service functions and benefits, directly expresses land greening and serves as the basic carrier of natural ecosystem service supply. Currently, vegetation changes are represented by land use/land cover (LULC) change, which are specifically manifested in the spatial-temporal pattern change and internal transfers of ecological land [12, 13]. However, the stability of ecological land, which reflects the characteristics of ecological land patterns and sustainability of ecological processes, and reveals the coordination between regional land use mode and natural ecosystem, is relatively scarce compared to dynamic research on ecological land, and mostly focuses on quantitative characteristics [14–17], while the explorations of functional stability are relatively weak. The land type stability of ecological land is the result of cumulative vegetation greening under the background of water availability, while functional stability reflects the impacts on the local ecosystem due to the composition and evolution of intrinsic vegetation structure. In recent years, remote sensing data has gradually become an important dataset for regional vegetation dynamic monitoring due to its continuity, timeliness, and other advantages [18]. Vegetation indices derived from remote sensing data can quantitatively express vegetation changes, among which the Normalized Difference Vegetation Index (NDVI), which has a significant linear correlation with vegetation distribution density, is widely used for monitoring ecological environmental conditions and evolutionary changes, such as desertification [19, 20]. Therefore, based on land use/land cover data and the NDVI index, it is possible to explore the land use types and functional stability of ecological land.

Research has shown that dynamic changes in soil moisture are important indicators of vegetation recovery. It has been found that vegetation greening may intensify inter-plant competition, exacerbate soil water deficit, and even trigger intra-plant water stress in northern China with water shortage [21–25]. Vegetation greening can lead to decreased soil moisture, which may worsen land desertification and cause irreversible degradation of the natural ecosystem. Therefore, understanding the dynamic changes in soil moisture is crucial for assessing the sustainability of the local ecosystem. Additionally, deep insights into the driving mechanisms of vegetation greening are essential for promoting ecosystem sustainable development. Natural factors such as climate change and nitrogen deposition significantly affect the growth and distribution of vegetation [2, 3, 26, 27], while human activities such as land management practices, ecological environmental protection projects and policies directly modify land cover conditions [4–6, 26]. Both natural environment and human activities have a profound impact on global vegetation greening. However, current research has mainly focused on investigating single driving factors, and it is still worth further exploration of the integrated effects of multiple factors and their dynamic changes.

Current research on vegetation restoration in large-scale areas has provided valuable insights into the hydrological effects, carbon balance, surface energy, and other ecosystem services [28–32], which are important for understanding the overall change trends of ecosystem services and large-scale ecological effects. However, it may not capture the spatial heterogeneity within the geographic region. Therefore, small-scale regional studies have become particularly essential. The Mu Us Sandy land (MUSL), one of the four major sandy lands in China, is located in the ecotone of farming and grazing in northern China, the lake alluvial plain depression between the Ordos Plateau and the Loess Plateau, and the intersection of the northern part of the East Asian monsoon region and the eastern edge of the westerly circulation. In addition, the community structure is relatively simple, dominated by shrubs and herbs [33], and there is a prominent conflict between humans and the environment, with a fragile ecological environment. Particularly since 2000, significant changes in vegetation conditions in the MUSL have occurred due to the strong influence of multiple major vegetation restoration projects and climate change [34–36]. The large-scale greening in a short period of time is the result of our country’s efforts to spend numerous human, financial and material resources, and how to maintain the stability of the greening results so that it can truly play the role of The Great Green Wall has become particularly important. Therefore, the paper takes the MUSL as an example, based on multi-source remote sensing data and these methods, such as stability index, quantitative remote sensing of vegetation, and Geodetector, the paper explores the sustainability of greening process in the study area from three aspects. From the changes of land use type and functional stability, firstly, LULC data is used to represent the greening stability of land classes (ecological land); And then, due to the obvious phenomenon of single dominance of vegetation communities in the MUSL (dominated by temperate grasslands), the functional greening stability is further explored through using MODIS NDVI data and derived FVC data. Additionally, the MUSL is located at the northern edge of the eastern monsoon region, so the water restriction factors for vegetation growth are particularly important to investigate. Finally, the influence mechanism of greening stability in the MUSL is explored.

## Materials and methods

### Study area

**Fig 1:**
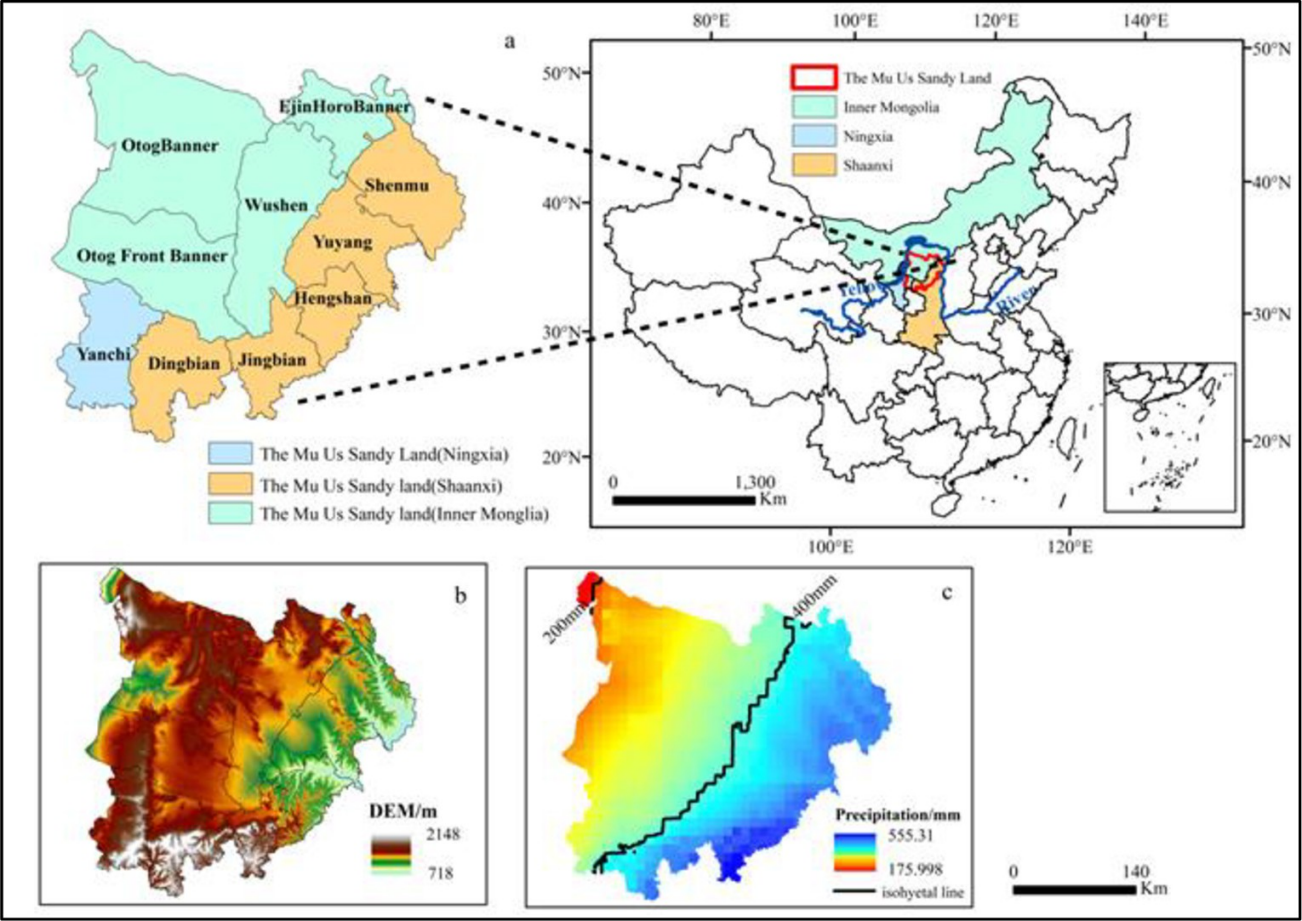
Location(a), landform(b) and distribution of average annual precipitation(c) in the study area.

The MUSL is located at the intersection of Inner Mongolia, Ningxia, and Shaanxi Province (37°27.5′N—39°22.5′N, 107°20′E—111°30′E) in northern China. It encompassed 10 counties (banners, districts), including OtogBanner, Otog Front Banner, Wushen Banner, and Ejin Horo Banner in Inner Mongolia; Shenmu County, Yuyang District, Hengshan County, Jingbian County, and Dingbian County in Shaanxi; Yanchi County in Ningxia [34] (Fig 1). The MUSL lies in the transition zone from the Ordos Plateau to the Loess Plateau, and it’s an important agro-pastoral ecotone of northern China. The terrain is generally high in the west and low in the east (Fig 1). Located at the edge of the East Asian monsoon and the Westerlies, the MUSL is characterized by temperate continental climate. The average annual precipitation ranges from 180 mm in the northwest to 560 mm in the southeast (Fig 1), which is largely concentrated in July—September, and the annual mean temperature reaches 7.4–9.0°C.

### Data sources

Land use/land cover (LULC) data were provided by the Data Center for Resources and Environmental Sciences, Chinese Academy of Sciences (https://www.resdc.cn/), which were generated by human-computer interaction interpretation of Landsat TM/ETM and Landsat 8 remote sensing image data [37]. The accuracy of the interpreted data was determined by several field surveys and random sampling checks performed over the years, and the results showed that the average classification accuracy exceeded 90%, indicating that the LULC data can be used as reliable base maps for subsequent analyses [38]. The data included five images taken in the years 2000, 2005, 2010, 2015, and 2020, with a spatial resolution of 30 m. The first-level classification included six categories: cultivated land, forest, grassland, water, construction land, and unused land [37]. Forest and grassland, with ecological service functions, were classified as ecological land. The LULC data were then resampled to 250 m. In addition to the LULC data, the global 30 m land cover product with a fine classification system in 2020 [39], and the 1:1000000 vegetation map of China [40] were also used in this paper.

MODIS NDVI (Normalized Difference Vegetation Index) was widely used in the study of vegetation cover change after water, cloud, and heavy aerosol processing [41]. The MOD13Q1 dataset provides NDVI data with a spatial resolution of 250 m and a temporal resolution of 16 days, and is from United States Geological Survey (USGS) (https://earthexplorer.usgs.gov/). In the study area, the annual maximum NDVI dataset for 2000, 2005, 2010, 2015, and 2020 were obtained through the maximum synthesis method in ArcGIS10.8, and then the annual average value of NDVI was calculated by means of average method. MOD13Q1_NDVI data has been widely used in the study area [42, 43]. The MOD17A3 dataset provides net primary productivity (NPP) data with a spatial resolution of 500 m for the year 2020, which is from USGS.

The FLDAS NOAH01_C_GL_M dataset provides soil moisture data (https://ldas.gsfc.nasa.gov/) with a temporal resolution of months and a spatial resolution of 0.1°×0.1°. This dataset assists in food security assessments in developing countries with scarce data, which contains information on many climate-related variables [44]. What’s more, the data were monitored by random sampling, the result showed that its spatial distribution was consistent with the observed data [45].

Meteorological data included variables such as average temperature (Tav), maximum temperature (Tmax), minimum temperature (Tmin), precipitation (Pre), sunhour, and average wind speed (Wind), and were provided by China Meteorological Information Sharing System (https://data.cma.cn). The monthly dataset comprises over 790 meteorological stations in China, of which a small number of missing values got completed by multiple linear regression, and then the Anusplin4.2 interpolation model [46] was used for spatial interpolation with a spatial resolution of 1000 m, finally extracted the meteorological elements distribution of the study area using mask extraction. The digital elevation dataset (DEM) with a spatial resolution of 90 m was provided by Shuttle Radar Topographic Mission (SRTM) [47], and the slope was calculated by DEM. The population density distribution data (Pop) were obtained from WorldPop (https://www.worldpop.org), with a spatial resolution of 1000 m. The kilometer grid data of the spatial distribution of GDP were derived from the Data Center for Resources and Environmental Sciences, Chinese Academy of Sciences in 2000, and from Zhao et al. [48] in 2020. The administrative boundary data is from the public version of Natural Earth (https://www.naturalearthdata.com/), and the sandy desert area is from Zhong [49]. All of the above data are publicly available, and are processed in ArcGIS10.8 for Desktop software platform.

### Methods

#### Spatial stability index based on LULC

From a LULC perspective, stability can be measured by the area proportion of the LULC types that remain unchanged within a region over a while. For this reason, the spatial stability of ecological land refers to the characteristics that ecological land maintains its land type attributes unchanged in the study area within a period [50, 51]. Therefore, the calculation method is as follows:

$$PSI=\frac{\sum_{i=1}^{n}A_{i}}{A}\times 100\%$$ (1)

Where *PSI* denotes the stability index of ecological land; *A*_*i*_ is the area of ecological land cover type *i* that maintains unchanged during the study period in the study area; *A* expresses the total area of all land types in the study area. The larger the *PSI* value, the higher the stability of regional ecological land; otherwise, the stability gets lower.

**Fig 2:**
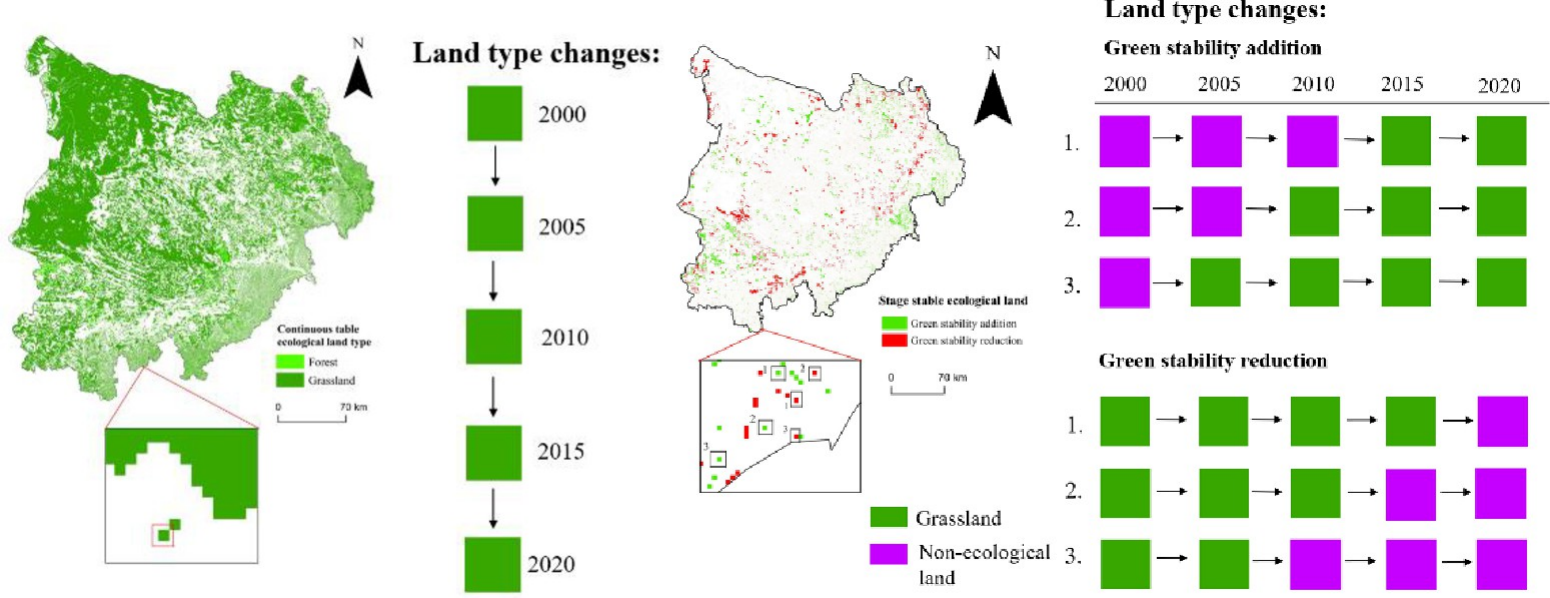
The example of continuous/stage stable ecological land.

If ecological land maintains its land type attributes unchanged during the study period in the study area, it can be called continuous stable ecological land, or greening stability [14–17]; Instead, if changes, it is classified as stage-stable ecological land, which can be divided into green stability addition and green stability reduction [17, 50, 51]. In five raster image data (2000/2005/2010/2015/2020), the land type of the grid unit has always been grassland (forest), which was continuous stable ecological land; If the land type was grassland (forest) in the first two (three/four) periods, and there existed no grassland (forest) of two continuous rasters in the last three (two/one) periods, which was called green stability reduction; If it was no grassland (forest) of two continuous rasters in the first (two/three) periods, and there existed grassland (forest) of two continuous rasters in the last four (three/two) periods, which was green stability addition. See Fig 2 for the conceptual concrete diagram, taking grassland as an example. The most basic and stable ecological land use—continuous stable ecological land, can guarantee the local ecological environment quality. The paper took it as the research subject to explore whether the ecological environment can be sustainable in the MUSL.

#### NDVI difference

Although NDVI can illustrate the overall change of regional vegetation, it is still a macro statistical index. To evaluate changes in vegetation density over time, NDVI difference of the same location and pixel needs to be employed [52], as expressed below:

$$\Delta NDVI={NDVI}_{T_{2}}-{NDVI}_{T_{1}}$$ (2)

Where ${NDVI}_{T_{2}}$ and ${NDVI}_{T_{1}}$ denote the pixel values of *T*_*2*_ and *T*_*1*_ (year) in the NDVI map within a period of time, respectively; Δ*NDVI* is the NDVI difference, and its value ranges from -2 to 2. The NDVI difference classification results are [52]: severe degeneration(Δ*NDVI*≤-0.15), moderate degeneration(-0.15<Δ*NDVI*≤-0.05), slight degeneration(-0.05<Δ*NDVI*≤0), slight improvement (0<Δ*NDVI*≤0.05), moderate improvement(0.05<Δ*NDVI*≤0.15), and extreme improvement(Δ*NDVI*>0.15).

#### Dune morphology recognition

The Mu Us Sandy Land is located on the northern edge of the East Asian monsoon region, the purpose of dune morphology recognition is to explore the soil water carrying capacity [53]. Meanwhile, different dune forms require different approaches to control desertification. Generally, vegetation cover is essential for controlling desertification. For these, the paper calculates dune morphology recognition. Based on the NVDI value, the pixel dichotomy model is employed to estimate vegetation coverage in the study area [54, 55], as expressed below:

$$FVC=\left(NDVI-{NDVI}_{soil}\right)/\left({NDVI}_{veg}-{NDVI}_{soil}\right)$$ (3)

Where *FVC* denotes vegetation coverage; *NDVI*_*soil*_ and *NDVI*_*veg*_ are NDVI values of the completely bare soil cover and dense vegetation cover, respectively.

Combined with the method proposed by scholars [54, 55], and the actual situation of vegetation coverage in the study area, a confidence interval of 0.5% was selected to intercept NDVI with a frequency of 99.5% as the upper threshold, and a frequency of 0.5% as the lower threshold. The upper and lower thresholds represent the values of *NDVI*_*veg*_ and *NDVI*_*soil*_ in the image, respectively. Here, dune morphology is the classification of mobility on surface material of hill under wind force, which is divided into bare (mobile) and vegetation cover (fixed, semi-fixed) according to fixing extent of dune [56]. According to the Technical Code of Practice on the Sandified Land Monitoring (GB/T 24255–2009) and Wu [56], the sandified land is divided into three categories: mobile dune (0<*FVC*<10%), semi-fixed dune (10%≤*FVC*<30%), and fixed dune (*FVC*≥30%).

#### Geodetector

The Geodetector model is a new statistical method for detecting spatial stratified heterogeneity and revealing the driving factors behind it, including four detectors: risk detector, factor detector, ecological detector, and interaction detector [57]. Factor detector and interaction detector are employed in this paper. For more information on the Geodetector, you can refer to the literature cited by Wang and Xu [57].

The driving factors affecting the greening stability of the study area, are discretized by the natural breakpoint classification method. However, due to the maximum sample size of the Geodetector being 32768 lines, a 2 km × 2 km fishnet with 10954 nets was created in the study area instead of a 1 km × 1 km fishnet used in previous studies with 44231 nets. NDVI is regarded as Y variable, and 10 driving factors are regarded as X variable (Tmin, Tmax, Tav, Sunhour, Pre, Wind, DEM, Slope, Pop, GDP) for research.

**Fig 3:**
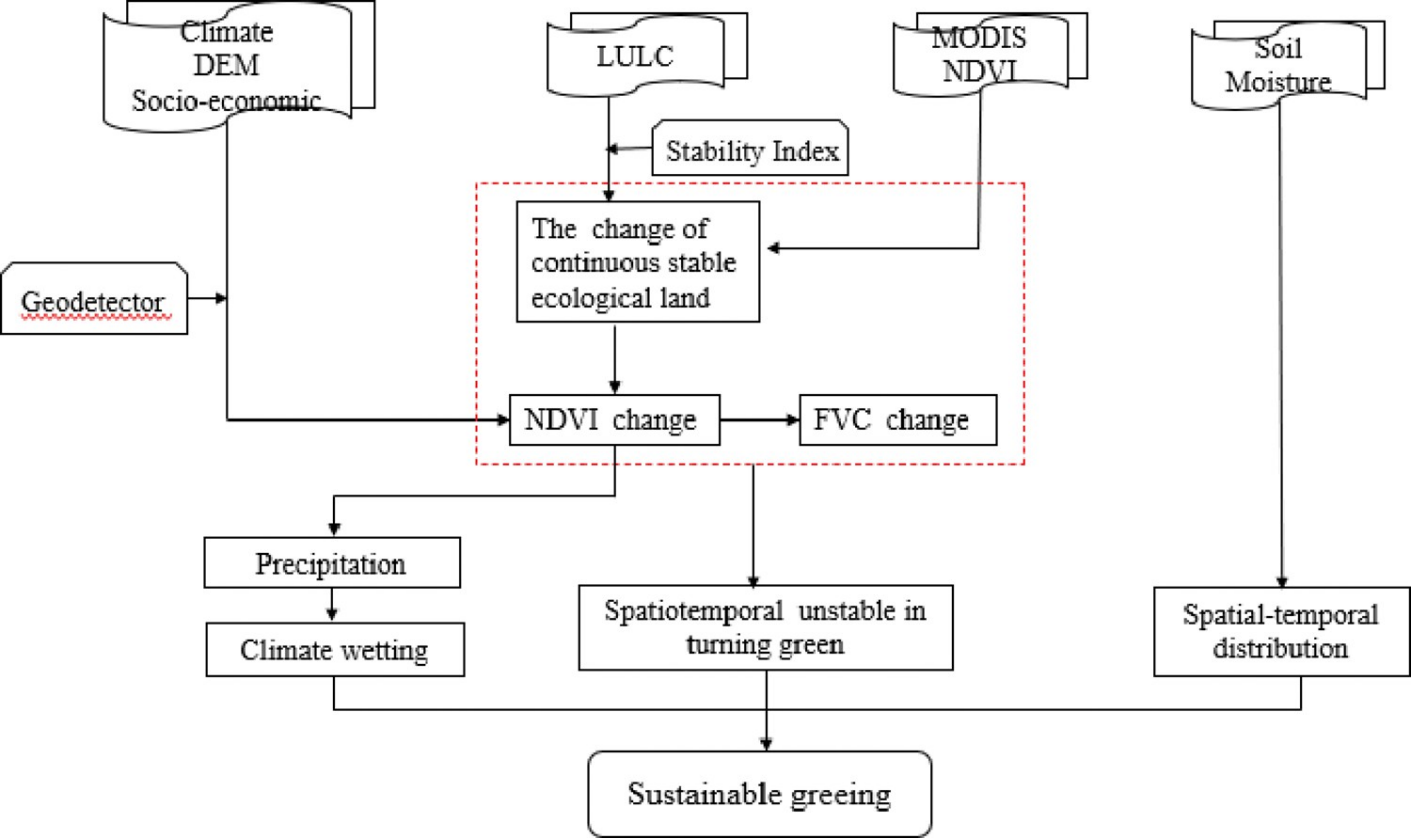
The technology roadmap for this study.

Therefore, based on the above data and methods, the research ideas of this paper can be seen in Fig 3.

## Greening stability process

### Changes in the stability of LULC types

Based on the five images of LULC data, ecological land area has shown a fluctuating increase over the past 20 years in the MUSL. The dominant land cover type in ecological land area was grassland, which made up more than 57% of the total area. Over the first 10 years, grassland grew by 0.86% but then declined by 1.5% from 2010 to 2020. Meanwhile, forest showed a slight increase, with an overall increase of 0.57%. The non-ecological land cover, such as cultivated land and unused land, both decreased by 0.72% and 0.45%, respectively. In contrast, construction land area climbed continuously from 0.47% to 1.73%, and water cover remained relatively stable (Table 1).

**Table 1:** Area proportion of ecological land in the Mu Us Sandy Land from 2000 to 2020/%.

	Ecological land		Non-ecological land			
	Forest	Grassland	Farmland	Construction land	Waters	Unused land
2000	2.46	57.89	15.32	0.47	1.23	22.63
2005	3.04	57.44	14.55	0.57	1.18	23.22
2010	2.94	58.75	14.81	0.89	1.07	21.53
2015	2.92	58.56	14.83	1.17	1.05	21.46
2020	3.04	57.25	14.60	1.73	1.22	22.17

**Fig 4:**
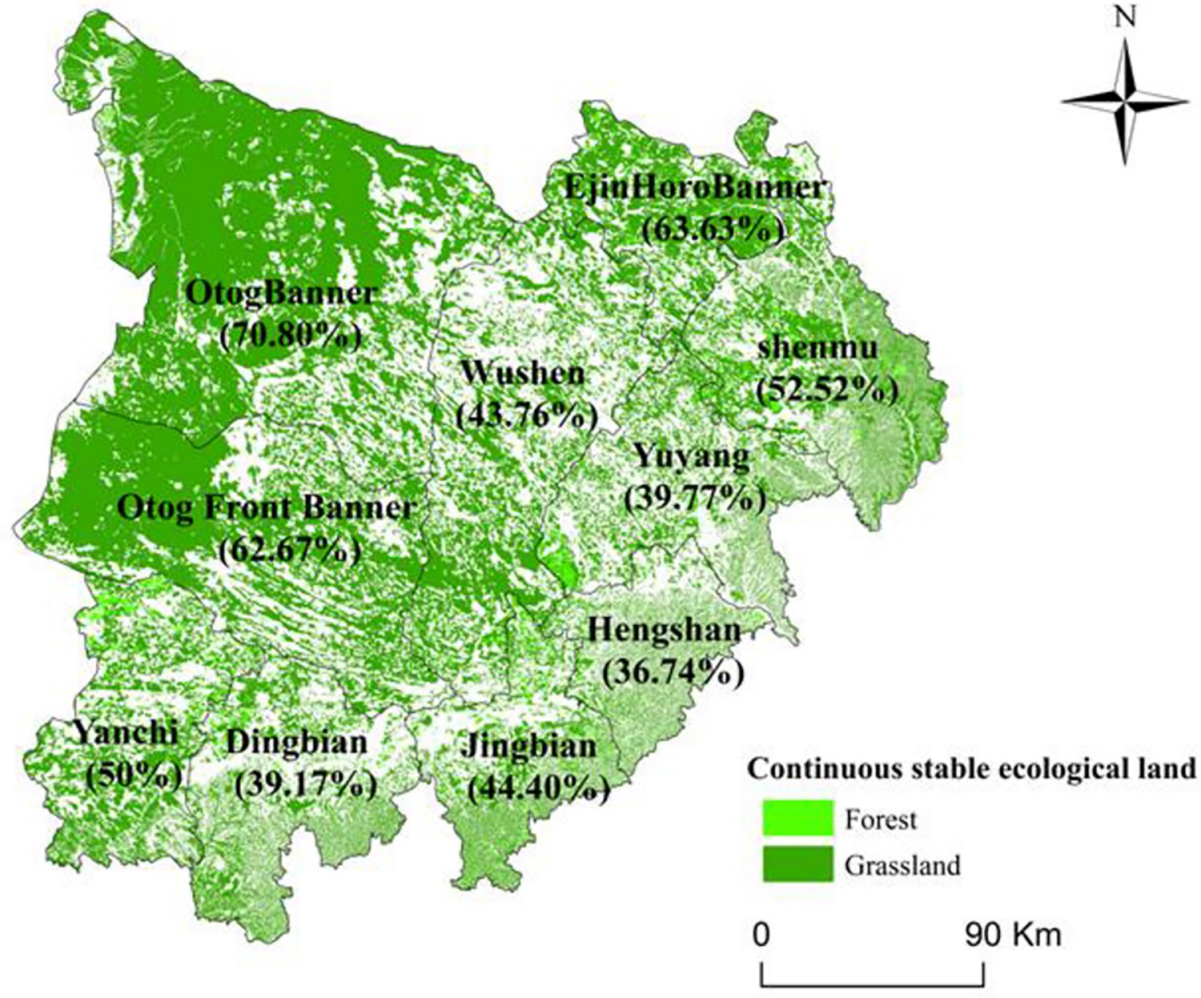
Spatial distribution and area proportion of continuous stable ecological land in the study area in 2000–2020.

Fig 4 illustrated the distribution of continuous stable ecological land in the study area, along with its area proportion in each county (banner, district) from 2000 to 2020. The data showed that the continuous stable ecological land area accounted for a significant portion of the study area, amounting to 54.94%. The majority of this land was comprised of grassland, which accounted for 53.04%, while forest was only 1.9%. The distribution pattern displayed low in the southeast and high in the northwest of the study area. The counties of OtogBanner, Otog Front Banner, and Ejin Horo Banner had the highest proportions of continuous stable ecological land, accounting for more than 60%.

### Changes in the functional stability

#### Vegetation change based on NDVI

**Fig 5:**
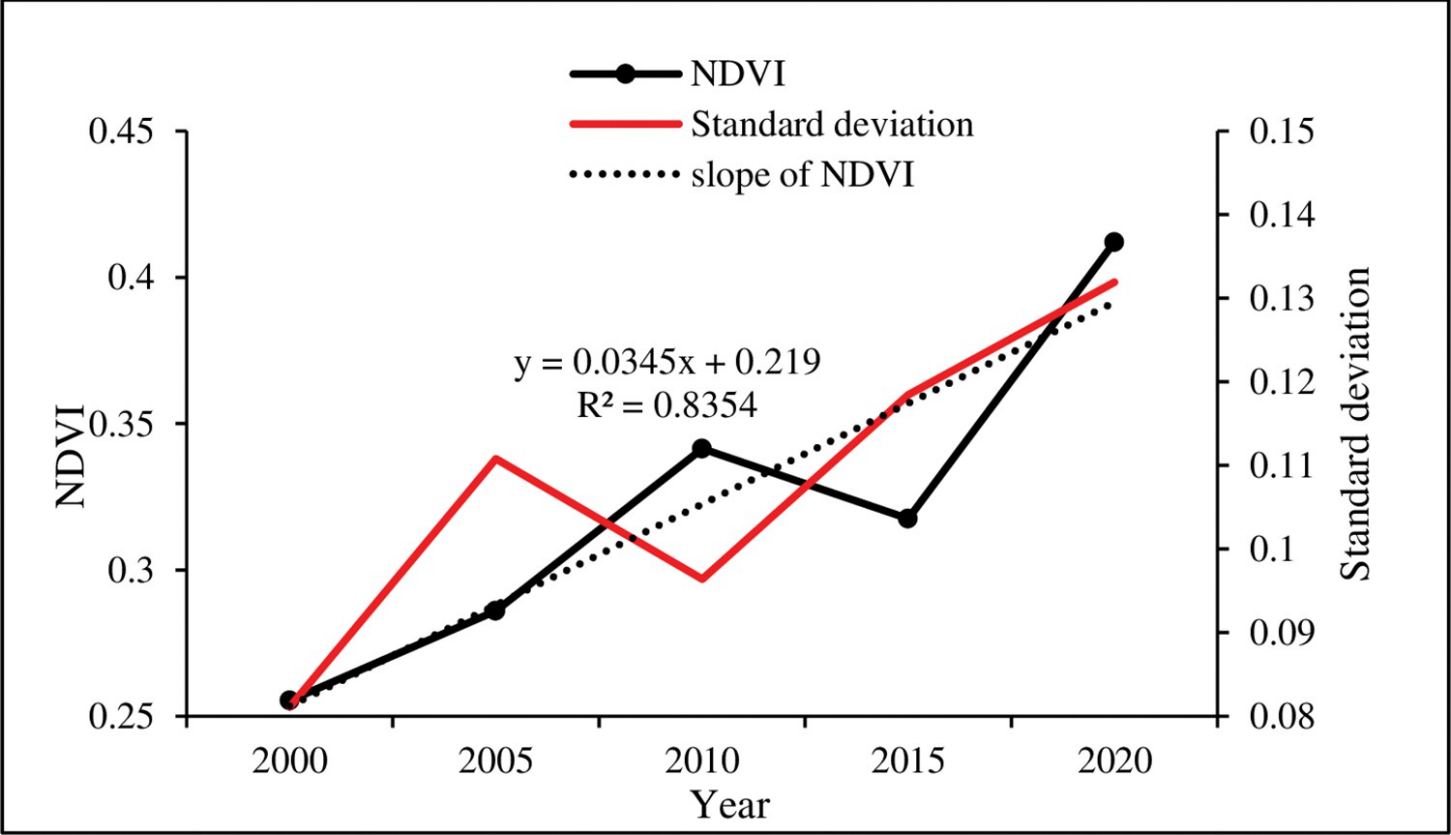
The NDVI change in the study area from 2000 to 2020.

**Fig 6:**
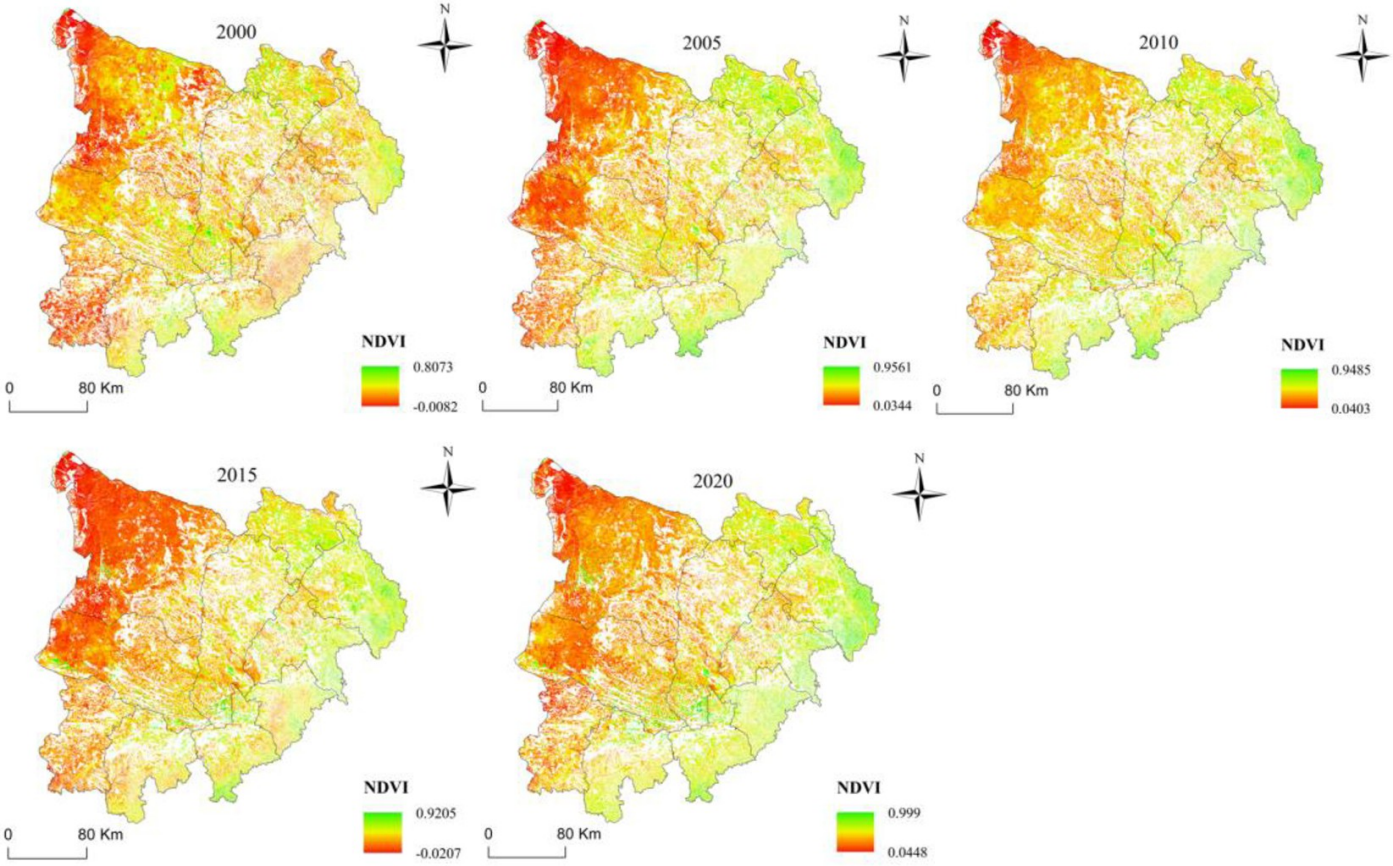
Spatial distribution of the NDVI of continuous stable ecological land in the study area in 2000–2020.

Figs 5 and 6 displayed the spatial-temporal distribution changes of NDVI of continuous stable ecological land in the MULS from 2000 to 2020. The result showed that vegetation NDVI expressed a fluctuating upward trend as a whole, with an average increase of 0.035 per year(p<0.01). However, this trend was not consistent and showed fluctuations during the study period. Vegetation NDVI continued to rise in 2000–2010, but it fell back in 2015 before rapidly increasing again. During this period, the coefficient of variation (CV) showed fluctuations, with the highest value observed in 2005 (0.38) and the lowest in 2010 (0.28) Furthermore, the study found that NDVI displayed high in the southeast and low in the northwest. OtogBanner, Otog Front Banner, and Yanchi have consistently been low-value distribution areas, with no significant changes in NDVI in the first two banners. In contrast, Yuyang, Shenmu, Hengshan, and Jingbian have always been high-value distribution areas, with a significant increase in NDVI over time.

**Fig 7:**
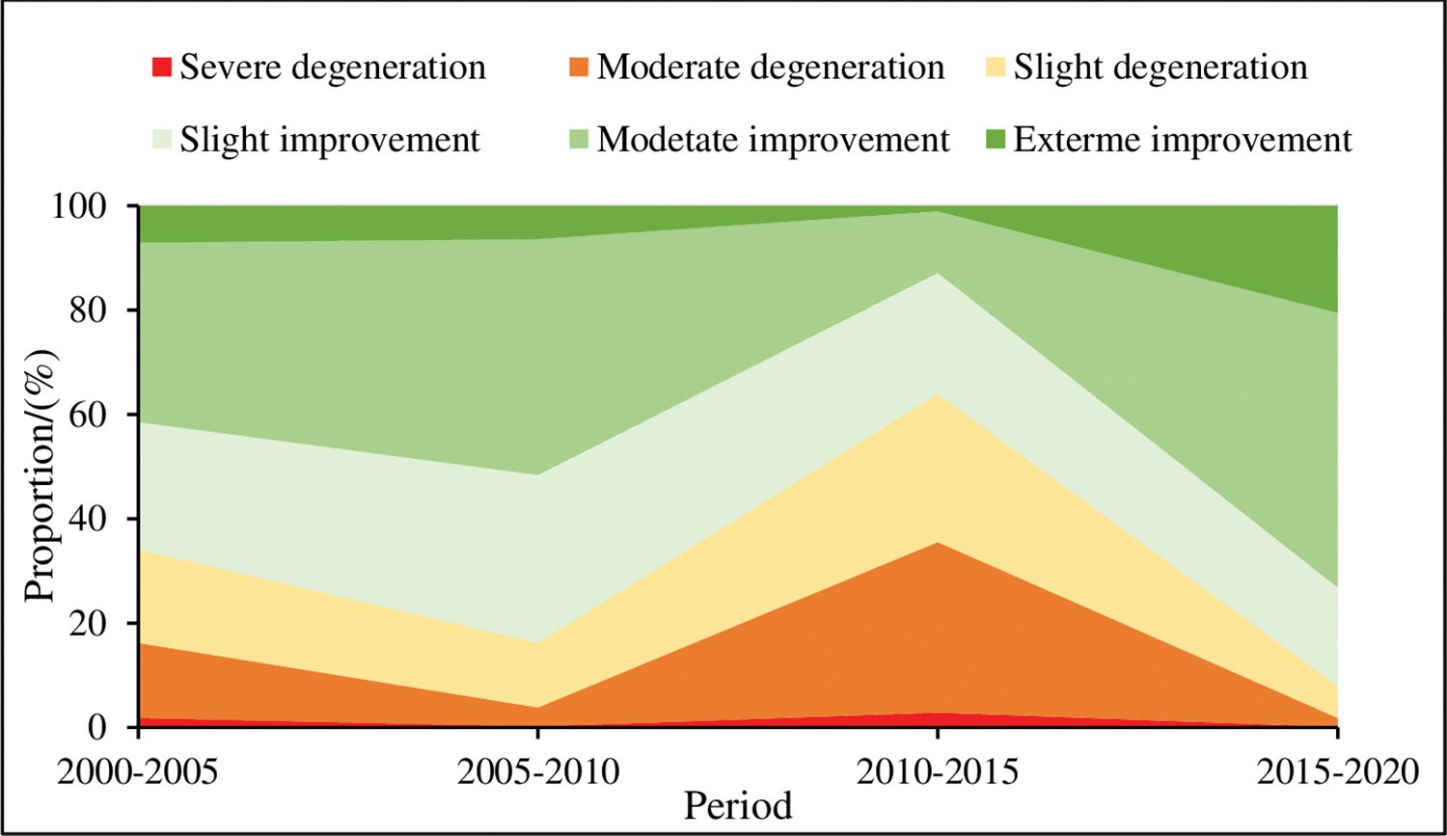
The different NDVI classes in the study area in 2000–2020.

**Fig 8:**
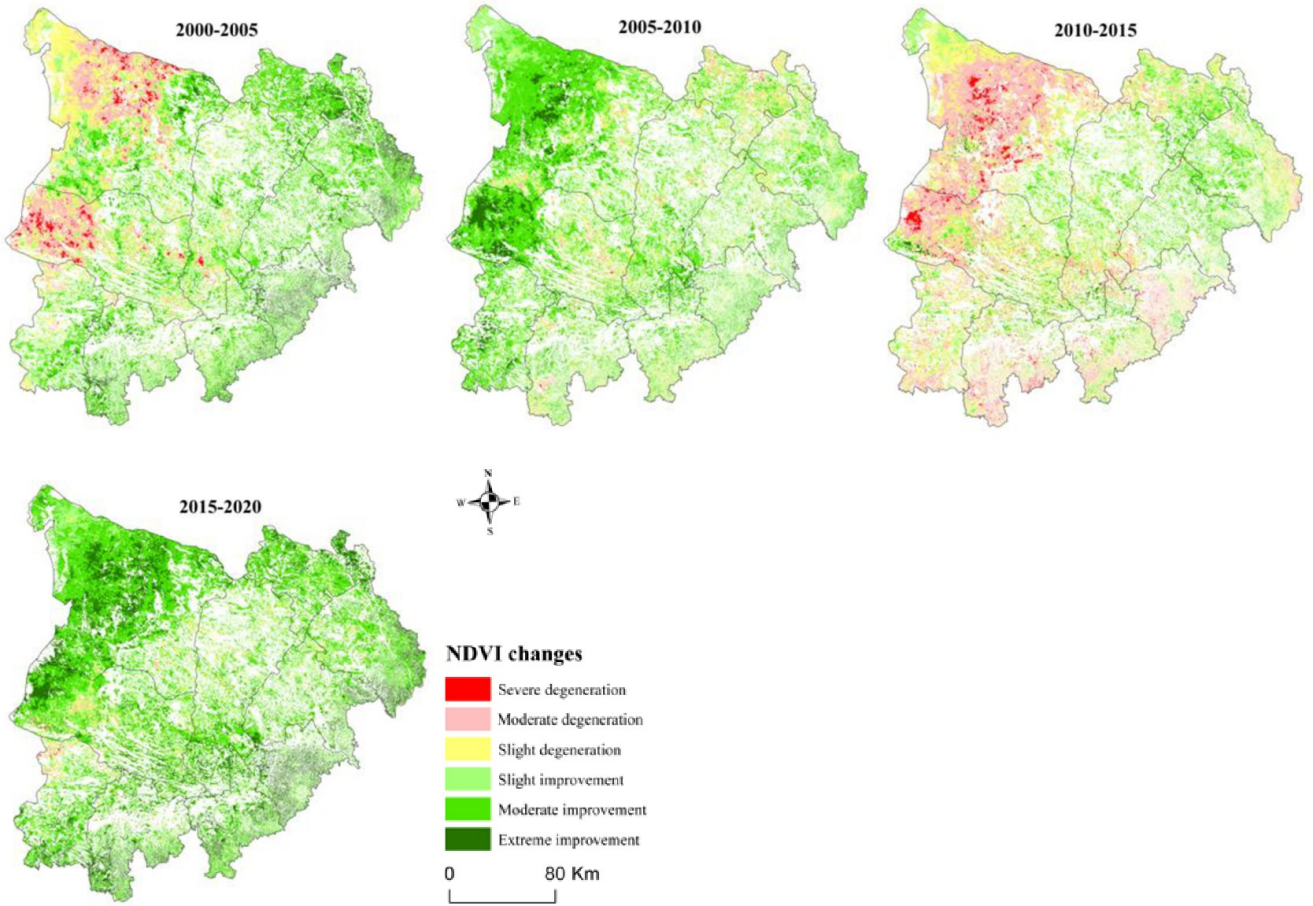
The spatial distribution of different NDVI classes in the study area in 2000–2020.

Based on the NDVI difference results presented in Figs 7 and 8, it can be concluded that vegetation has been a general improvement trend in 2000–2010 in the MUSL. The area proportion of vegetation improvement grew from 65.82% to 83.83%, with moderate improvement being the dominant type. At the same time, vegetation degradation decreased from 34.18% to 16.17%. However, during 2010–2015, vegetation degradation got more severe, and the area proportion increased to 63.94%, mainly with moderate degradation. But in 2015–2020, vegetation improvement jumped to 92.06%, with moderate improvement accounting for 52.63% and extreme improvement reaching 20.53%. Regarding spatial distribution, the northwest part of OtogBanner and Otog Front Banner were mainly affected by vegetation degradation in 2000–2005, accounting for about 60% of the Banner area respectively, while slight and moderate vegetation improvement was observed in the rest of the study area. In 2010, vegetation degradation area had been improved in the northwest part of OtogBanner and Otog Front Banner, while vegetation improvement area had been slightly degraded in Ejin Horo Banner, and not much had changed in Wushen and Yuyang. By 2015, vegetation had degraded overall, especially in the northwest of OtogBanner and Otog Front Banner, where was moderate and slight degradation, and so was the southern MUSL (Yanchi, Dingbian, Jingbian, and Hengshan), while Ejin Horo Banner had remained relatively stable. By 2020, vegetation had been improved comprehensively, except for slight degradation in the central part of Otog Front Banner, the northwest of Yanchi, and the central part of Wushen Banner. On the whole, the study suggested that vegetation degradation area has declined, and vegetation improvement has grown indicating that vegetation conditions were developing towards a virtuous cycle, and the process of sandification can be somewhat curbed. However, the alternating changes in NDVI also indicated that vegetation development remains unstable in the northwestern MULS.

#### Dune morphology change based on FVC

**Fig 9:**
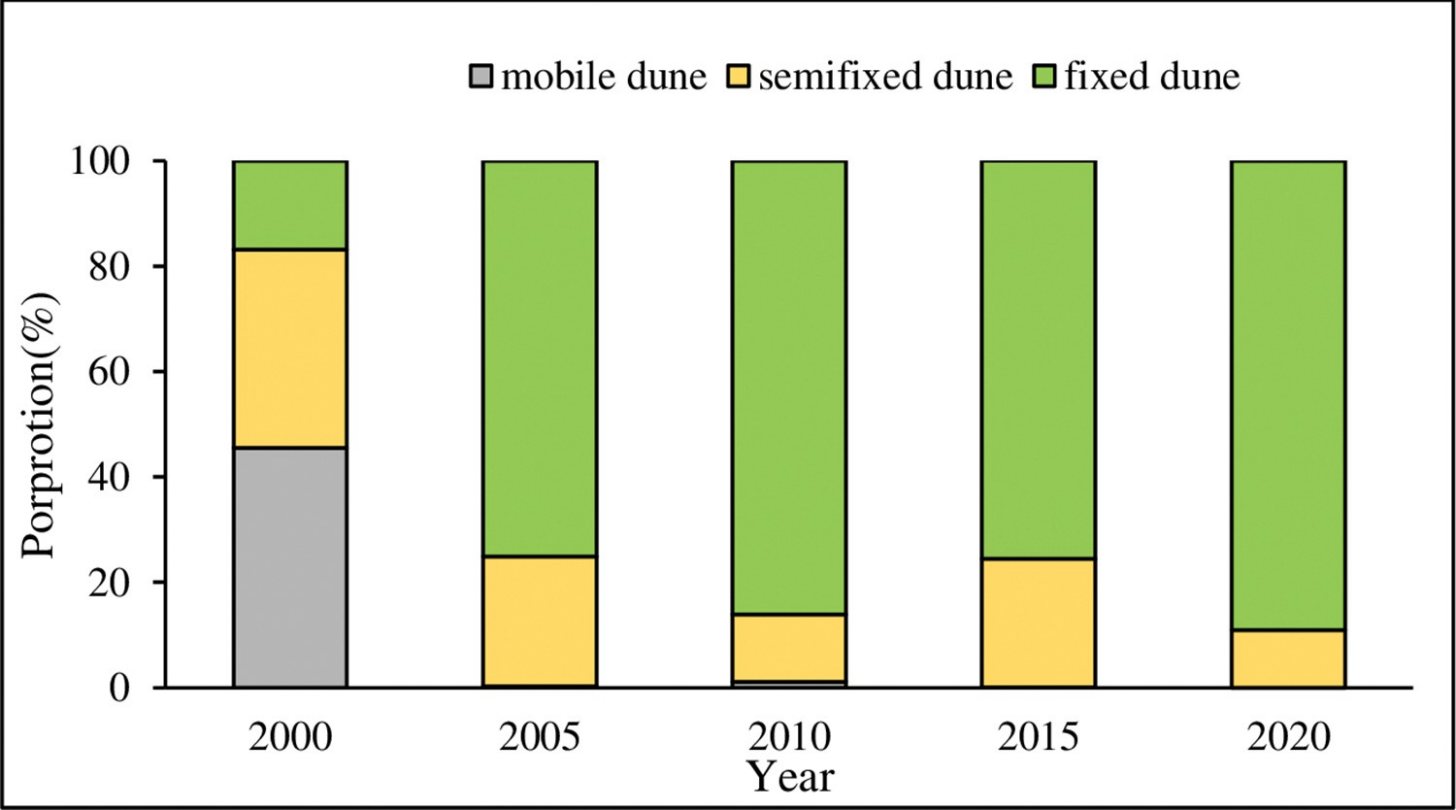
The proportion of the different dune types in the study area in 2000–2020.

In the sandy desert area of the study area, FVC was calculated based on the NDVI value of the continuous stable ecological land, to investigate the spatial-temporal variation patterns of different dune types. Fig 9 found that fixed dunes were the dominant type of dune in the MUSL. However, there were significant internal fluctuations in different dune types from 2000 to 2020. Mobile dunes displayed a decreasing trend overall, with a rapid decrease in 2000–2005 followed by a slow rise and then a continuous decline. Semi-fixed dunes also appeared a declining trend, in which its area proportion rapidly fell from 37.7% to 12.8%, followed by fluctuated and then decreased to 11%. Fixed dunes generally showed an increasing trend, with a significant rise in proportion from 16.8% to 86.1%, followed by a decrease, and then a further increase to 89%. One noteworthy phenomenon was the rapid increase in fixed tunes between 2000 and 2005, which was attributed to human activities such as the implementation of the “grazing prohibition, rest grazing, and rotating grazing” policy, the Grain for Green Program, and the Beijing-Tianjin Sand-storm Source Project. These initiatives were aimed at restoring vegetation coverage in the MUSL within a short time.

**Fig 10:**
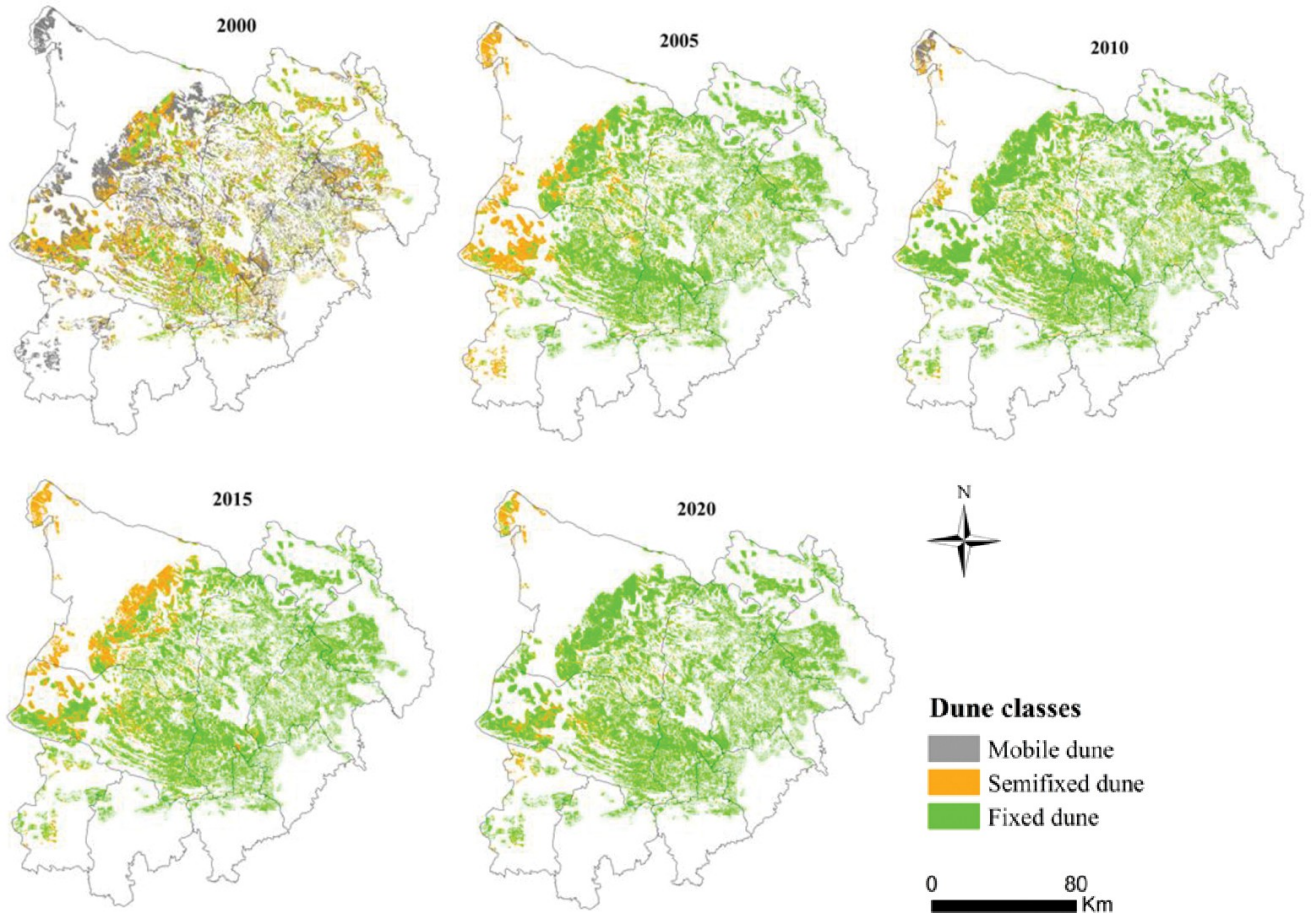
The spatial distribution of different forms of dunes in the study area in 2000–2020.

**Fig 11:**
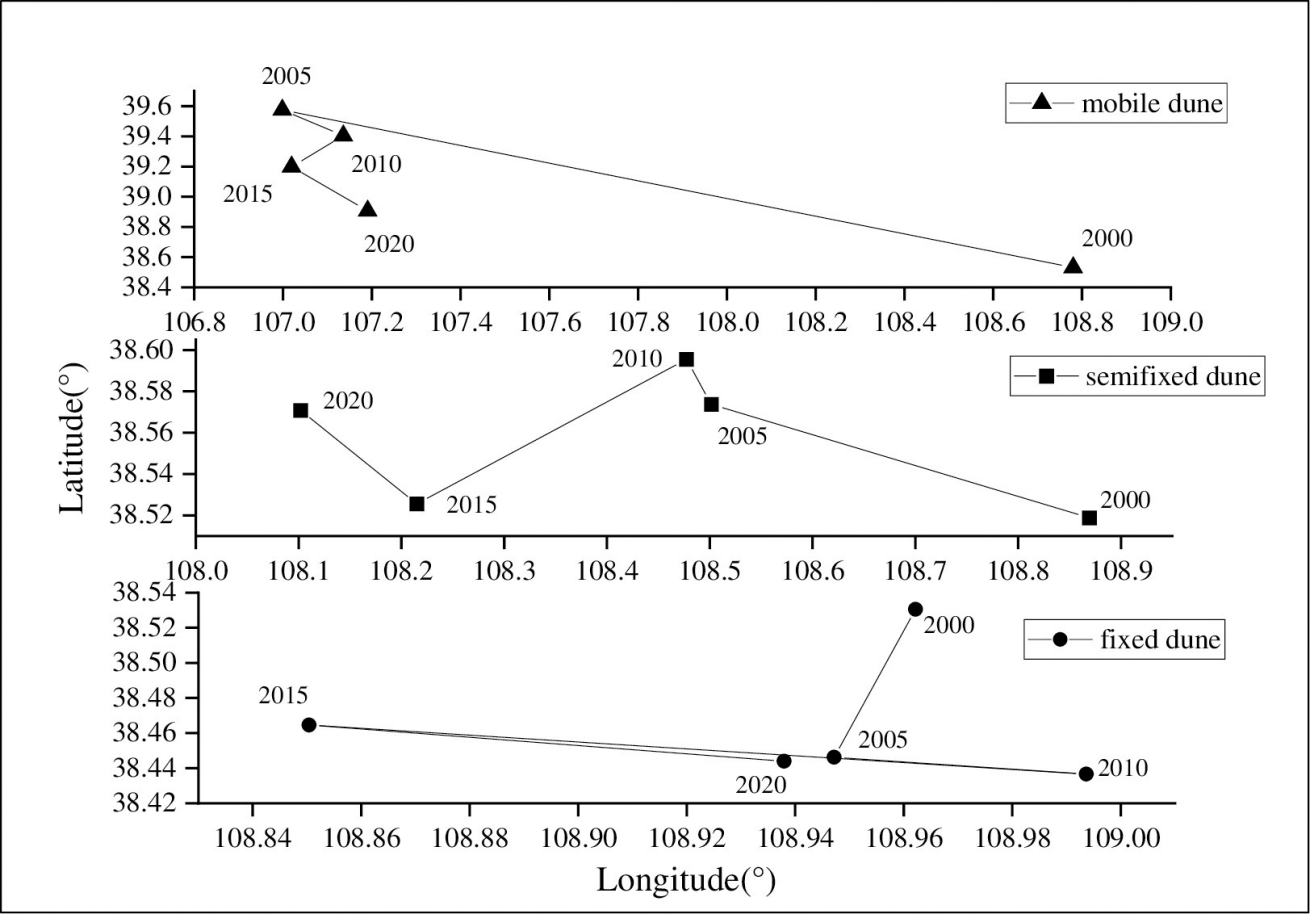
The gravity center migration of different types of dunes in the study area in 2000–2020.

Combining the distribution (Fig 10) and gravity center transfer (Fig 11) of different dune types, the paper was to comprehensively research its spatial distribution. Mobile dunes were dispersed in the study area in 2000, concentrated in the western part of the sandy area in 2005, and the northwest edge of OtogBanner in 2010, after that nearly disappeared. As for semi-fixed dunes, they gradually dispersed to sporadic distribution in the western part of the sandy area in 2000–2010, and then expanded in 2015, with obvious distribution in OtogBanner and Otog Front Banner, after that gradually shrank to the northwest edge of OtogBanner and the center part of Otog Front Banner. Fixed dunes had always been widely distributed in the central part of the sandy area. In accordance with gravity center transfer, different dune types migrated westward on the whole, but the differences were that mobile dunes shifted the furthest, and moved southward after 2005; Followed by semi-fixed dunes, which only moved to the southwest during 2010–2015, and moved to the northwest in other years; The final was fixed dunes, which presented a more complicated shift process of southwest-southeast-northwest-southeast.

### Soil moisture change

**Fig 12:**
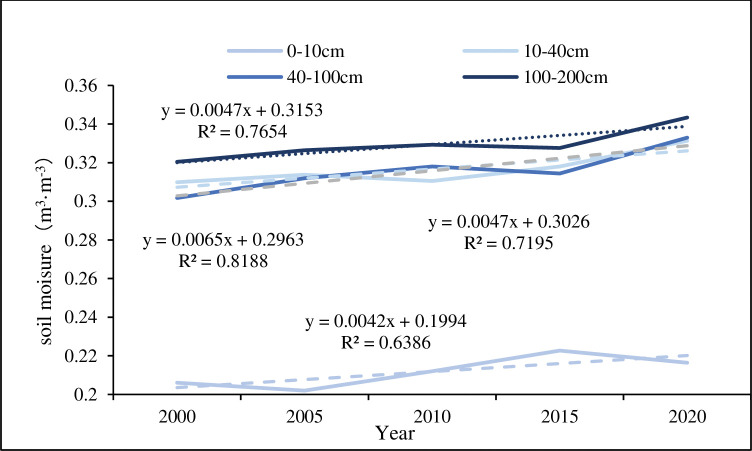
Mean soil moisture at different depths in the Mu Us Sandy Land from 2000 to 2020.

Soil moisture is the main source of water uptake by plants in arid and semi-arid regions [58]. Due to the problem of data acquisition, the data used in the paper had a relatively coarse spatial resolution, which can only provide a rough estimate of the overall soil moisture in the MULS, but cannot distinguish its changes in different dune types. On the whole, the data showed that soil moisture at the underground depths of 0-200cm has had an increasing trend in the past 20 years (Fig 12), indicating that vegetation restoration has played a positive impact on the local environment to a certain extent. Viewed from different underground depths, soil moisture tended to increase roughly with the underground depths, that is to say, the deep soil moisture was higher than that of the shallow (Fig 12). Additionally, there exerted spatial distribution differences in shallow and deep soil moisture. The spatial distribution pattern of soil moisture was high in the southeast and low in the northwest at 0–10 cm underground depth (S1(A) Fig), which was consistent with the distribution of precipitation (Fig 1) and NDVI (Fig 6) in the MULS. Meanwhile, Zhang and Wu. [22] concluded that NDVI had a strong positive correlation with shallow soil moisture, and Chen et al. [58] believed that the shallow soil moisture got closely related to precipitation. What’s more, compared with the surrounding areas, soil moisture was always less than that in the southern part of the MULS (southeastern Yanchi, most areas of Dingbian and southern Wushen) and most areas of Ejin Horo Banner at 10–200 cm underground depth (S1(B)-S1(D) Fig), the reason why differences in soil moisture distribution were that dune geomorphology [59], sand-fixing vegetation evolution [60], and the evolution of different dune types [61].

### The analysis of influencing factors

**Fig 13:**
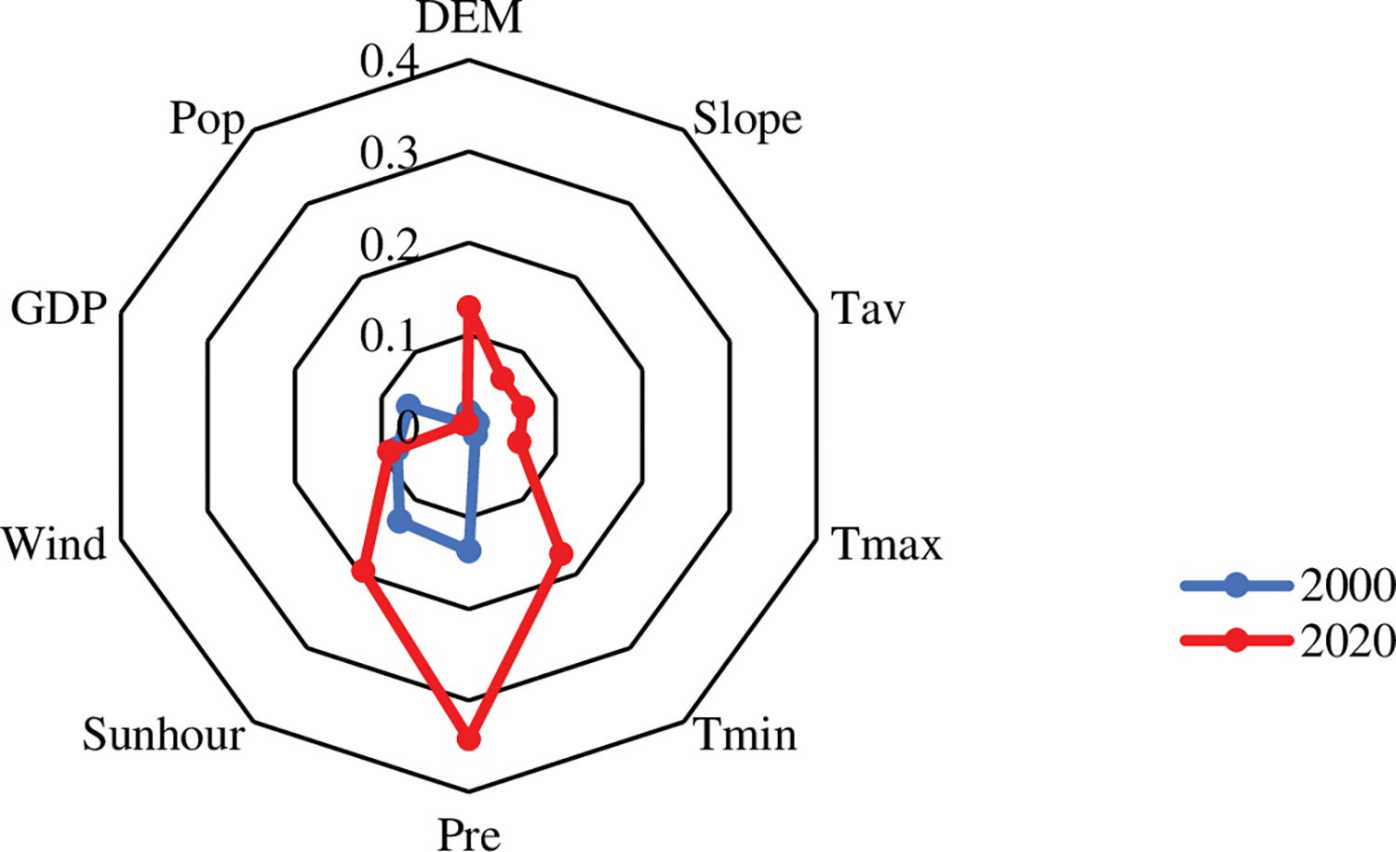
The factor detection results of NDVI in the study area in 2000–2020.

Through factor detector, the influence of driving factors on the NDVI of continuous stable ecological land was explored from natural factors (topography and climate) and social factors (population and economy) (Fig 13). The results exhibited that, except for social factors, natural factors exerted a significant impact on NDVI (p<0.01), and its explanatory power has increased over time. Precipitation, sunhour, and wind were found to have the strongest explanatory effects on greening stability (p>0.08), with precipitation being particularly prominent. Additionally, DEM and Tmin also increased significantly.

**Fig 14:**
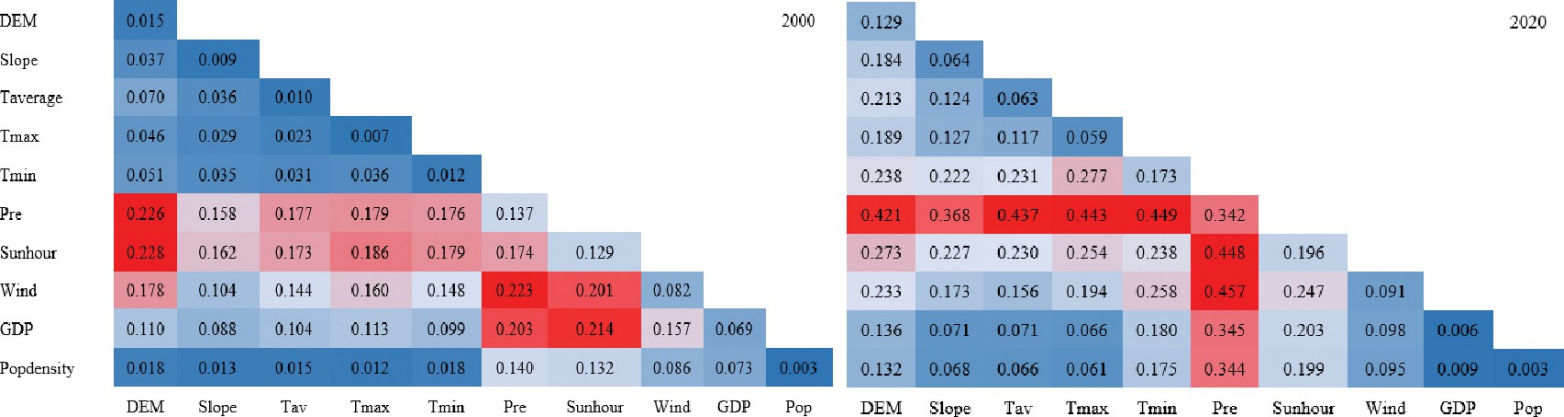
The interaction detection results of NDVI in the study area in 2000–2020.

The paper suggested that NDVI is affected by multiple factors and that there exert interactive relationships among the influencing factors. The interaction detector showed nonlinear or double-factor enhancement (Fig 14), both of which can help to strengthen the interpretation of NDVI changes. Furthermore, due to precipitation having a strong persuasive power to NDVI, the interaction with other factors displayed a strong explanatory power, which was significantly higher than other interaction variable combinations.

## Discussion

### The importance of grassland restoration to the local ecological environment

Adopted the first-level classification system of LULC established by Liu [62], the paper classified grassland and forest with ecological service functions into ecological land to explore the overall ecological environment of the study area. However, to understand the distribution of vegetation types more accurately, the paper employed land cover and vegetation data based on a fine vegetation classification system. According to the vegetation map of China (1:1000000), the study area was dominated by herbaceous vegetation, accounting for 70% of the area, which included grassland, meadow, and herbaceous swamp vegetation type groups. Shrub vegetation, which included shrub and desert vegetation type groups, accounted for 7.4% of the study area (S2 Fig). In light of vegetation types distribution and NPP data in 2020 (S3 Fig), the average NPP value is 184.04 gC/m^2^ in the MUSL, with low net primary productivity. There were significant differences in NPP among different vegetation types. (1) Grassland and sparse vegetation were the dominant vegetation types, accounting for 41.43% and 24.50% of the study area, with NPP value of 189.82 gC/m^2^ and 172.81 gC/m^2^, respectively; (2) Herbaceous cover, which occupied 1.27%, NPP value was 235.83 gC/m^2^; (3) Other vegetation types, including forest, accounted for less than 1%. However, forest had the highest NPP value, with needle-leaved forest and broadleaves forest having NPP value of 252.16 gC/m^2^ and 276.73 gC/m^2^ respectively, while wetland had the lowest NPP value of 137.06 gC/m^2^. At the same time, the distribution pattern of NPP was generally high in the surrounding area and low in the middle. To be specific, the NPP values were low in the eastern part of OtogBanner, most areas of Wushen, and the western part of Yuyang, while high in Dingbian and Southern Jingbian. Studies have manifested that the woody plant cover was positively correlated with the average annual precipitation in China [63]. The MUSL, lying in arid and semi-arid areas, has experienced an increase in precipitation in the past 40 years [64]. However, the aridity has not changed much, resulting in a small area of forest. Therefore, the paper suggested that although forests have the highest NPP, compared with the large area of grassland, which made less contribution to local greening and ecological restoration [65, 66].

### The vulnerabilities in greening stability process

The paper discussed the conversion of ecological land to non-ecological land from 2000 to 2020 (Table 2). Green addition and green reduction areas in each period accounted for no more than 4% of the study area, which was lower than that of the continuous greening stability (54.94%). Meanwhile, the net change of greening areas grew positively from 1.08×10^4^ ha to 10.53×10^4^ ha before 2010, while after 2010, it decreased negatively from 1.84×10^4^ ha to 10.39×10^4^ ha, showing a reverse greening phenomenon. The above data indicated that there has exerted vulnerability and instability in the greening stability process of the MUSL, and the impact has deepened. The conversion of ecological land and non-ecological land, of which were cultivated land and unused land, got the most frequent; What’s more, ecological land encroachment by construction land cannot be ignored, all of which were the direct cause of the vulnerability in greening stability process. Due to special geographical location, fragile and harsh ecological environment, relatively slow economic development, backward production mode, the coexistence of large-scale industry and small-scale agriculture, weak awareness of environmental protection among residents, and other reasons, all these led to the contradiction between ecological construction and economic development, and tense human-land relationships, which were the root cause of the vulnerability in greening stability process [34, 36]. Despite the fragility of greening stability, the MULS displayed a trend of humidification (4.4772 mm/yr) in the past 20 years (S4 Fig), which contributed to the overall greening of continuous ecological land (Fig 5), stable green in the eastern MUSL, and expansion of greening stability area in the western MUSL (Fig 6). The paper suggested that this trend, coupled with the strengthening or continuous intensification of the Westerlies and the East Asian monsoon [67, 68], may promote stable greening in ecological land conversion areas in the MUSL in the future.

**Table 2:** The conversion area between ecological land and non-ecological land from 2000 to 2020/ha.

		2000–2005	2005–2010	2010–2015	2015–2020
Green addition	cropland-ecological land	95239.44	59724.9	12539.79	62707.50
(Non-ecological land convert into ecological land)	water-ecological land	3102.93	14699.07	2382.66	2388.51
	Construction land-ecological land	90.81	3560.58	423.72	17583.03
	unused land-ecological land	38632.14	156844.17	28343.61	85865.49
	Total area	137065.32	234828.72	43689.78	168544.53
Green reduction	ecological land- cropland	29211.84	76252.59	14800.5	45655.11
(Ecological land convert into non-ecological land)	ecological land-water	1739.88	2157.57	721.71	17727.84
	ecological land- construction land	5905.71	19303.38	18610.83	45108.00
	ecological land- unused land	89413.02	31835.97	27984.60	163973.61
	Total area	126270.45	129549.51	62117.64	272464.56
Increase(+)decrease(-)		10794.87	105279.21	-18427.86	-103920.03

### The importance of precipitation on greening stability

Based on the information provided, it appeared that precipitation significantly affected vegetation greening stability in the MUSL (Fig 13). S5 Fig provided data on the distribution of the green stability addition and reduction areas in the study area over the past 20 years. The data in S5 Fig found that green stability addition area was 26.11×10^4^ ha, accounting for 3.01% of the study area; Green stability reduction area was 29.37×10^4^ha, accounting for 3.38%, which showed that green stability reduction area was slightly higher than green stability addition area in the past 20 years. Furthermore, the distribution of green stability addition and reduction areas in arid and humid zones suggested that in arid and semi-arid areas, green stability reduction area was larger than green stability addition, while in semi-arid areas, green stability reduction area was less than green stability addition. Specifically, green stability addition, nearly 49.48% were distributed in semi-arid areas (annual precipitation is 200-400mm), 50.48% in semi-humid areas (annual precipitation is 400-800mm), only 0.04% in arid areas; Green stability reduction accounted for 51.85% in semi-arid areas, 46.42% in semi-humid areas, and 1.73% in arid areas. The results indicated that precipitation plays an important positive role in vegetation restoration to a certain extent, which is consistent with previous studies [34].

Precipitation is the only supply source of soil water in the MUSL [69]. The soil water carrying capacity of vegetation is an important basis to measure whether sand-fixing vegetation can be successfully reintroduced to the area, which can be defined as the maximum coverage that limited soil moisture can bear sand-fixing vegetation [59, 70]. Li et al. [71] proposed the eco-hydrological threshold for arid and semi-arid climate zone (annual precipitation is 250–500 mm), the average herbaceous coverage rate was 55% ([34, 63]), woody vegetation coverage rate was 17% ([10, 34]), and soil water carrying capacity was 8% ([4.3–14.1]). At the same time, Liu et al. [72] concluded that water resource carrying capacity (ecology/living/production) has generally shown a downward trend since 2000 in the MUSL (Inner Mongolia), and predicted that if the current development remains unchanged, it will be deteriorated by 2030. Based on the summary of previous research and the actual situation of the study area, we found that the eco-hydrological threshold should be considered to serve as a guide for managing the artificial sand-fixing vegetation ecosystem; What’s more, appropriate vegetation density and drought-tolerant vegetation types should be taken into account to avoid excessive planting damaging to soil water carrying capacity in arid and semi-arid areas. Only in this way can the ultimate goals of ecological restoration and regional sustainable development be realized.

### Reasonable control of human factors to prevent secondary sandification

Given data availability and other reasons, only population and economic indicators were selected for human activities in the paper, both of which displayed an insignificant impact on vegetation greening in the MUSL (Fig 13). However, a large number of studies have shown that human activities exerted a significant influence on vegetation [34, 36, 73, 74]. The MUSL is located in the transition zone of farming and grazing, and the number of livestock (cattle and sheep) has increased in the past 20 years [73], but vegetation cover has still turned green, the phenomenon presented was closely related to the policy of “grazing prohibition, rest grazing, and rotating grazing” promulgated since 2000 73]. At the same time, with the implementation of a massive number of national key projects since 2000, such as soil and water conservation ecological project, the Grain for Green Program, natural forest protection project, key counties of ecological constriction, vegetation improvement areas have gradually taken a dominant position in the MUSL, and the extreme improvement area has grown significantly (Fig 7). In addition, vegetation improvement areas showed an overall increasing trend in the western pastoral areas (Fig 8). All these indicated that the positive effects of environmental protection policies and projects on vegetation restoration. Furthermore, due to the differences in site conditions, in the middle and northern parts of the sandy land devoted to grazing, a supporting structure of grass breeding and livestock species, and an efficient breeding and animal husbandry structure focusing on grass and dairy industries should be established; In the eastern and southern regions dominated by agriculture, a grain-saving animal husbandry structure focusing on feed grain and meat production should be built, and feeding sheds should be constructed vigorously. Coupled with the increasing number of livestock, increased vegetation carrying capacity. Animals not only have shrubs and herbs, such as *Agriophyllum squarrosum* (Linn.) Moq., *Achnatherum splendens* (Trin.) Nevski, *Avena fatua* L., *Lespedeza bicolor* Turcz., *Helianthus tuberosus* L., *Pugionium cornutum* (L.) Gaertn., *Hippophae rhamnoides* L., *Caragana kors hinskii* Kom., *Artemisia desertorum* Spreng., but can be processed into feed by silage and microstorage technology. On one hand, it promoted the rejuvenation and renewal of adult vegetation; On the other hand, it solved the problem of insufficient feed, reduced the cost of breeding, and improved the enthusiasm of the farmers to return farmland to forest and grass [75].

## Conclusion

From 2000 to 2020, the proportion of continuous stable ecological land area in MUSL remained over 50%, accounting for a relatively high proportion. NDVI showed a significant increasing trend of 0.035/a, and a decreasing spatial distribution pattern from southeast to northwest. Although vegetation change was unstable, it was mainly vegetation improvement, distributed in the eastern part of the sandy land, while the vegetation change in the western region is extremely unstable. At the same time, the mobile dunes almost disappeared, the proportion of semi-fixed dunes decreased to 11%, gradually shrinking to the western part of the sandy area, while the fixed dunes soared to 89%, concentrated in the middle of the sandy area. In addition, the overall soil moisture showed an increasing trend. Based on land types and functional stability, and soil moisture change, the results show that the greening process of MUSL keep stable in the past 20 years, and the ecological environment is developing a good and orderly direction. What’s more, according to the results of factors affecting the continuous stable ecological land, natural factors, especially precipitation, exerted a significant impact on the stable greening, but the impact of human activities cannot be ignored. Finally, the research time scale is 20 years. Based on the actual situation, the research results are obtained from multi-source remote sensing data rather than field research, but the study results are still scientific to a certain extent. We will refine field research in the future studies.

## Supporting information

S1 Fig The average soil moisture at the underground depth of 0-10cm (a), 10-40cm(b), 40-100cm(c), and 100-200cm(d) in the study area in 2000–2020.
(ZIP)

S2 Fig The distribution of natural vegetation of the study area in 1:100,0000 vegetation distribution maps of China. (TIF)

S3 Fig The spatial distribution of vegetation types and their NPP value in the study area in 2020. (ZIP)

S4 Fig Annual precipitation characteristics in the Mu Us Sandy from 2000 to 2020. (PDF)

S5 Fig The spatial distribution of green stability addition/reduction in arid and humid areas. (TIF)
