# Supplementary figures and images for: Research on the sustainability of "greening" process in the Mu Us Sandy Land based on the spatiotemporal stability of ecological land
Source: PLoS One. 2024 Feb 6;19(2):e0292469. doi: 10.1371/journal.pone.0292469 (PMC10846700; doi:10.1371/journal.pone.0292469)

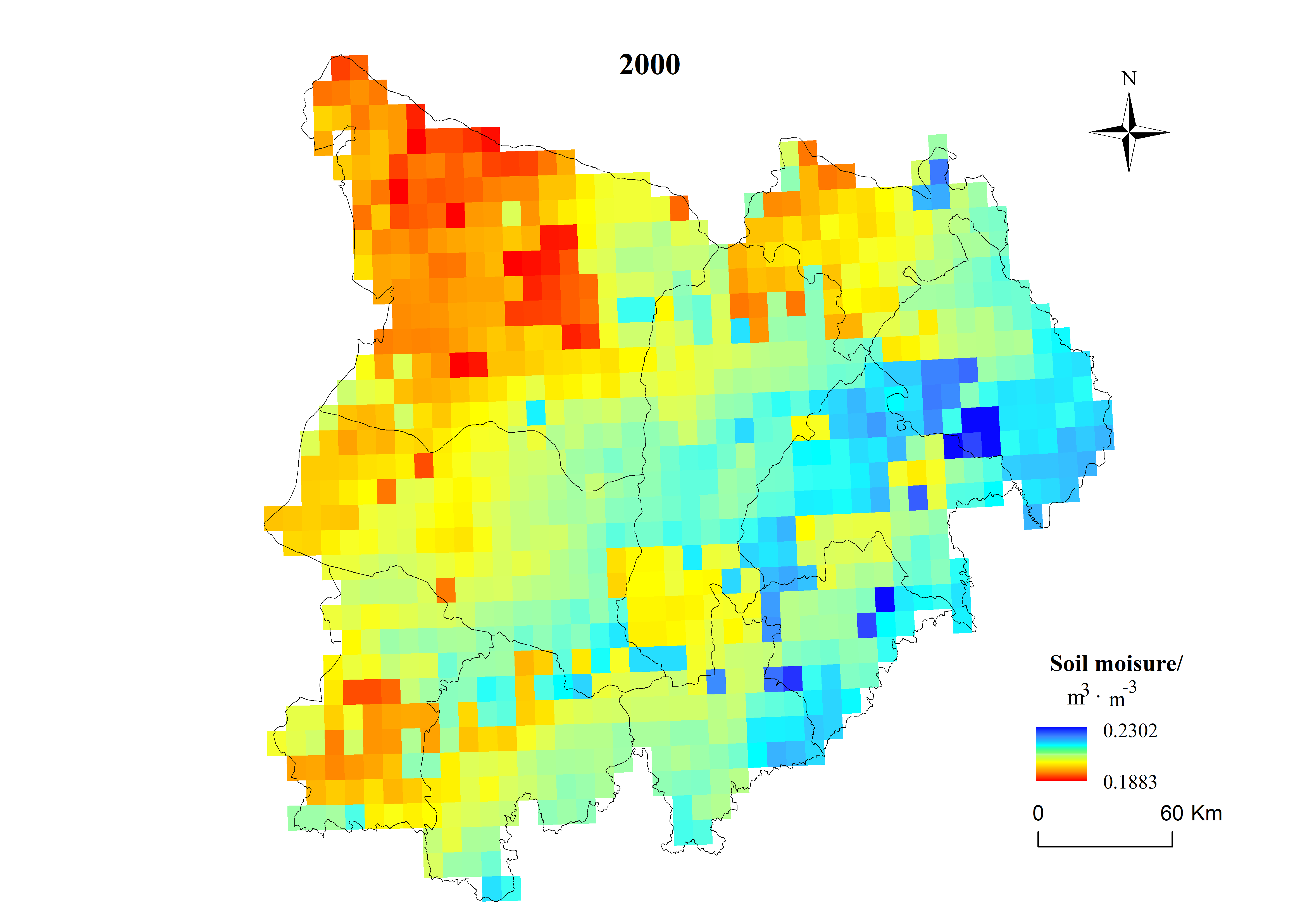

Supplement: S1 Fig — The average soil moisture at the underground depth of 0-10cm (a), 10-40cm(b), 40-100cm(c), and 100-200cm(d) in the study area in 2000–2020. (ZIP) [file pone.0292469.s001.zip › S1 Fig/(a)/2000.tif]

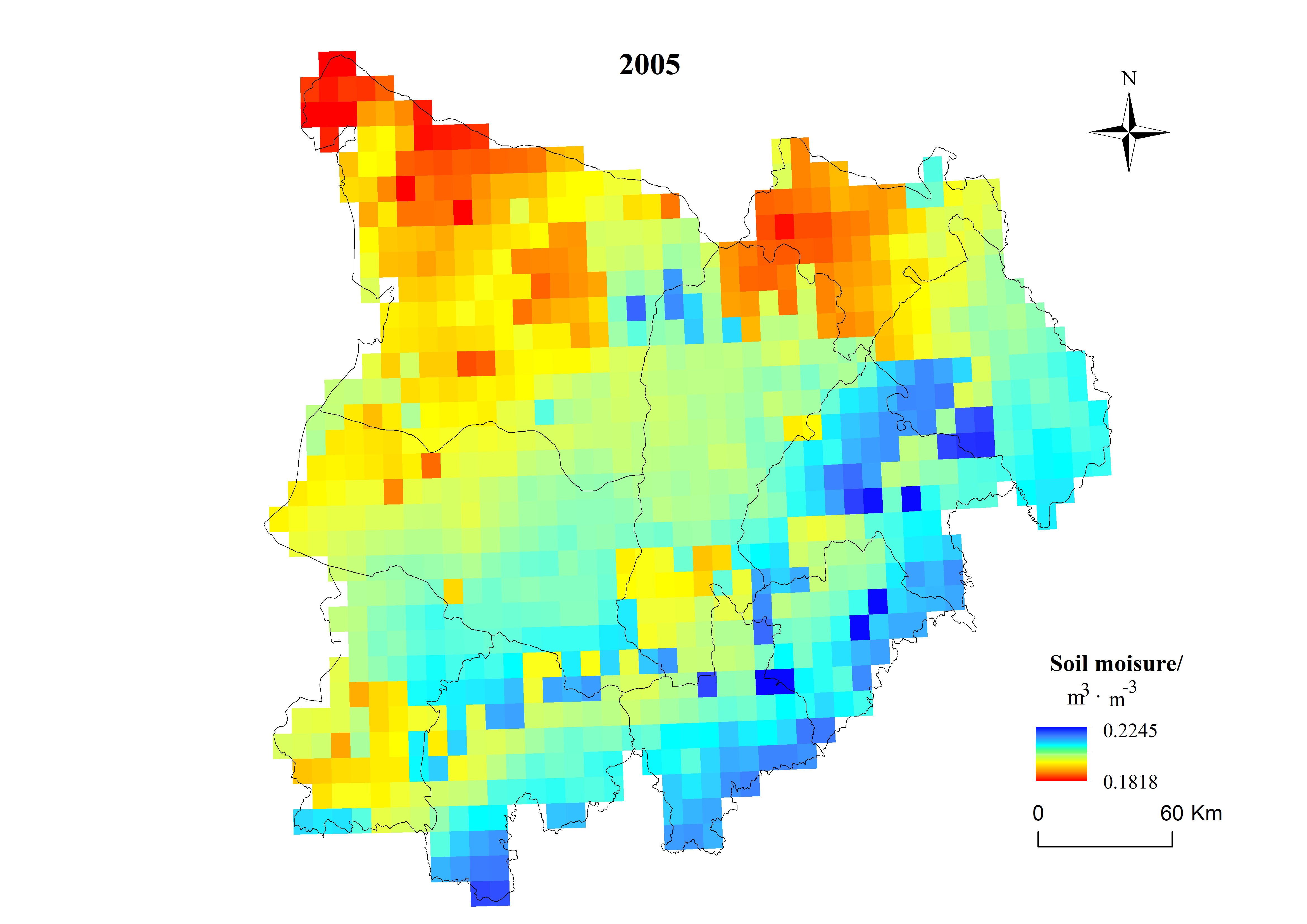

Supplement: S1 Fig — The average soil moisture at the underground depth of 0-10cm (a), 10-40cm(b), 40-100cm(c), and 100-200cm(d) in the study area in 2000–2020. (ZIP) [file pone.0292469.s001.zip › S1 Fig/(a)/2005.tif]

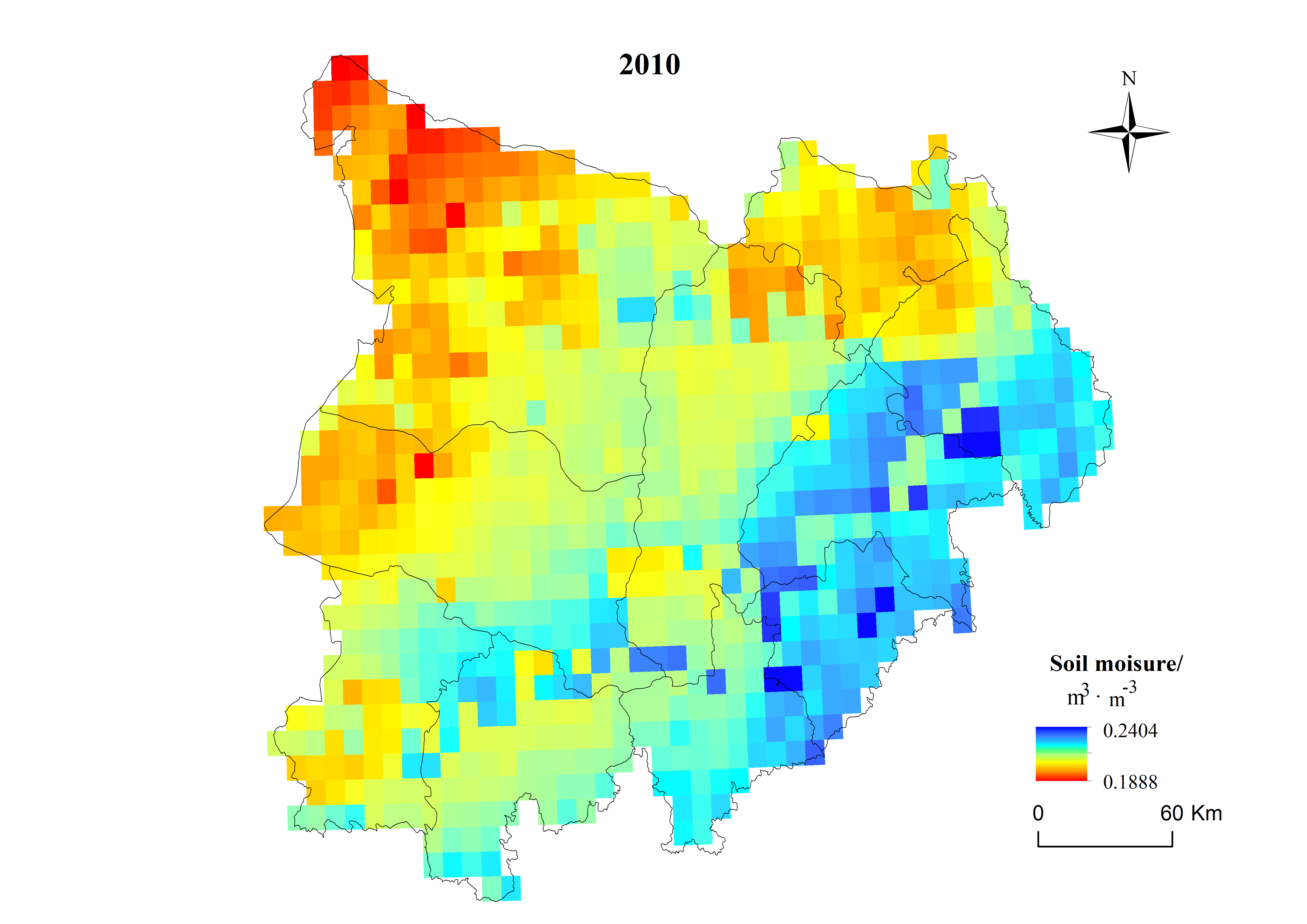

Supplement: S1 Fig — The average soil moisture at the underground depth of 0-10cm (a), 10-40cm(b), 40-100cm(c), and 100-200cm(d) in the study area in 2000–2020. (ZIP) [file pone.0292469.s001.zip › S1 Fig/(a)/2010.tif]

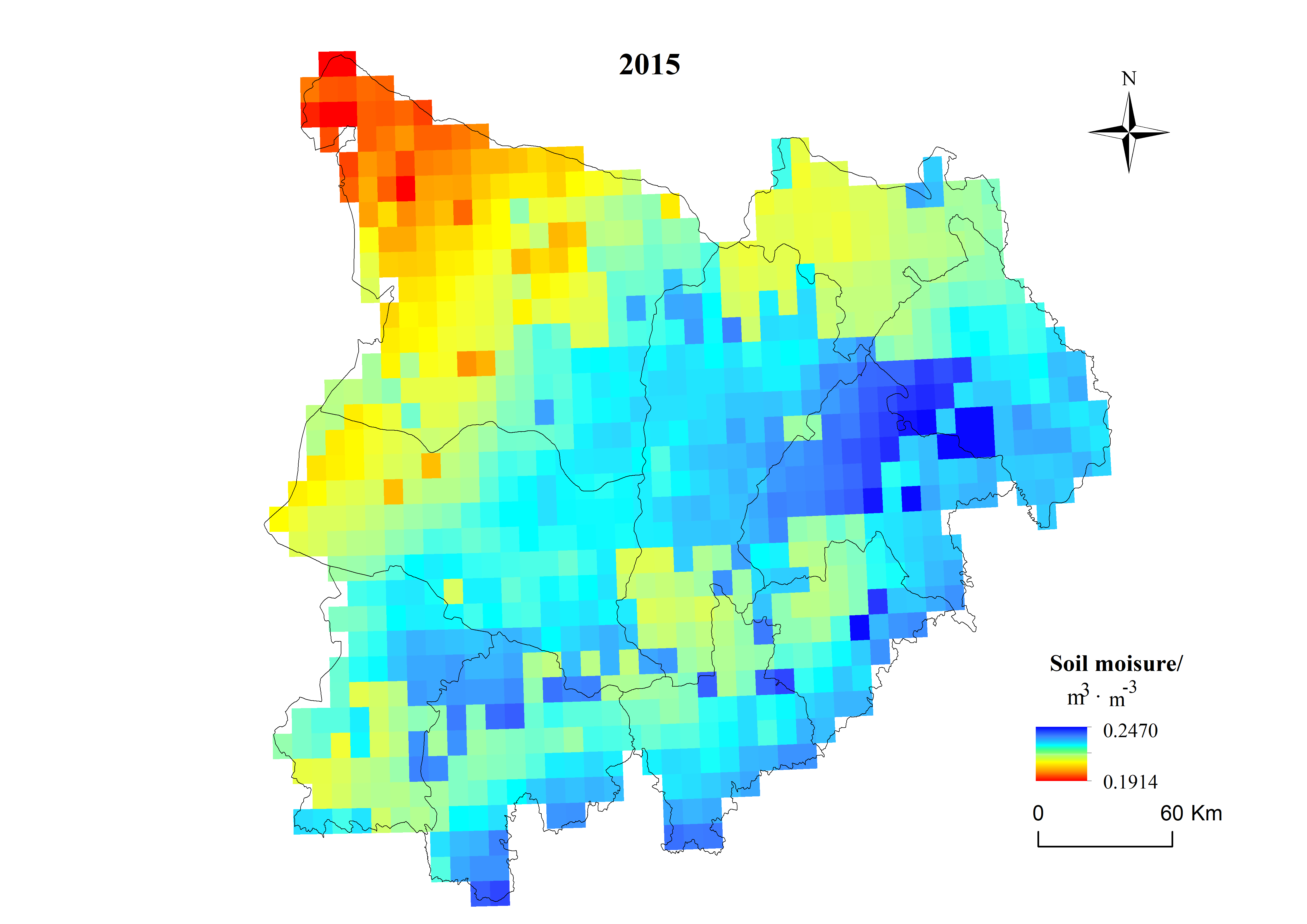

Supplement: S1 Fig — The average soil moisture at the underground depth of 0-10cm (a), 10-40cm(b), 40-100cm(c), and 100-200cm(d) in the study area in 2000–2020. (ZIP) [file pone.0292469.s001.zip › S1 Fig/(a)/2015.tif]

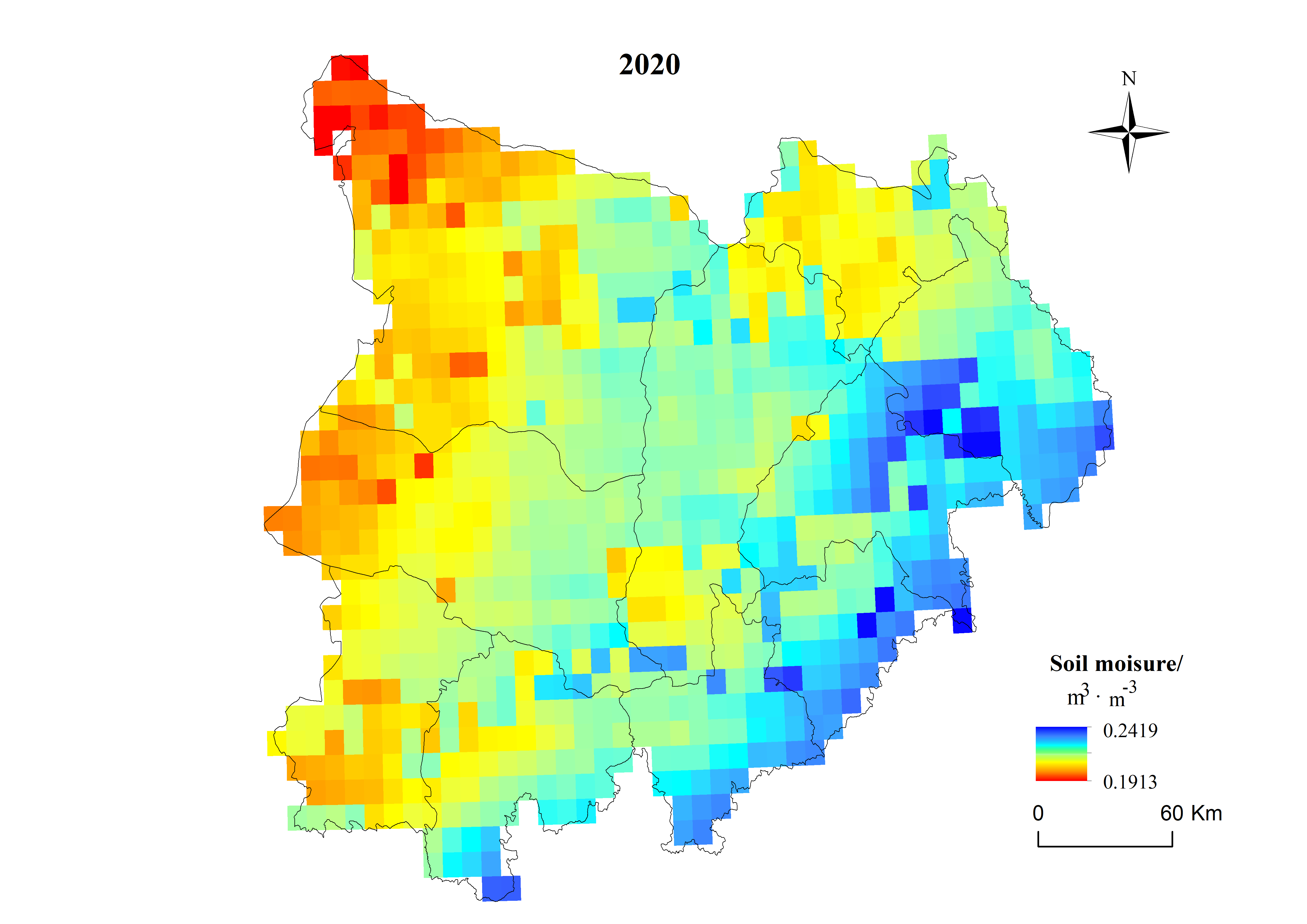

Supplement: S1 Fig — The average soil moisture at the underground depth of 0-10cm (a), 10-40cm(b), 40-100cm(c), and 100-200cm(d) in the study area in 2000–2020. (ZIP) [file pone.0292469.s001.zip › S1 Fig/(a)/2020.tif]

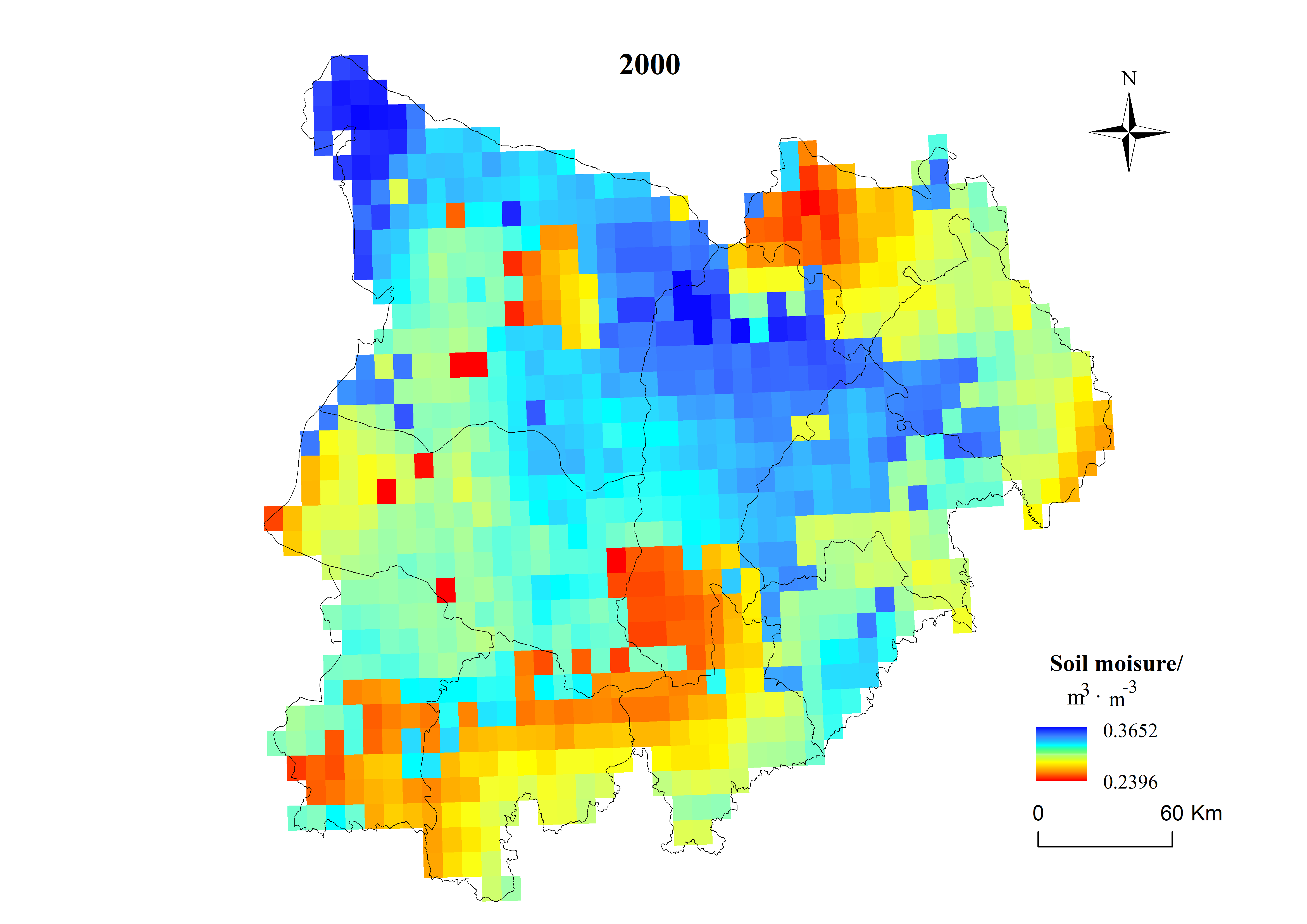

Supplement: S1 Fig — The average soil moisture at the underground depth of 0-10cm (a), 10-40cm(b), 40-100cm(c), and 100-200cm(d) in the study area in 2000–2020. (ZIP) [file pone.0292469.s001.zip › S1 Fig/(b)/2000.tif]

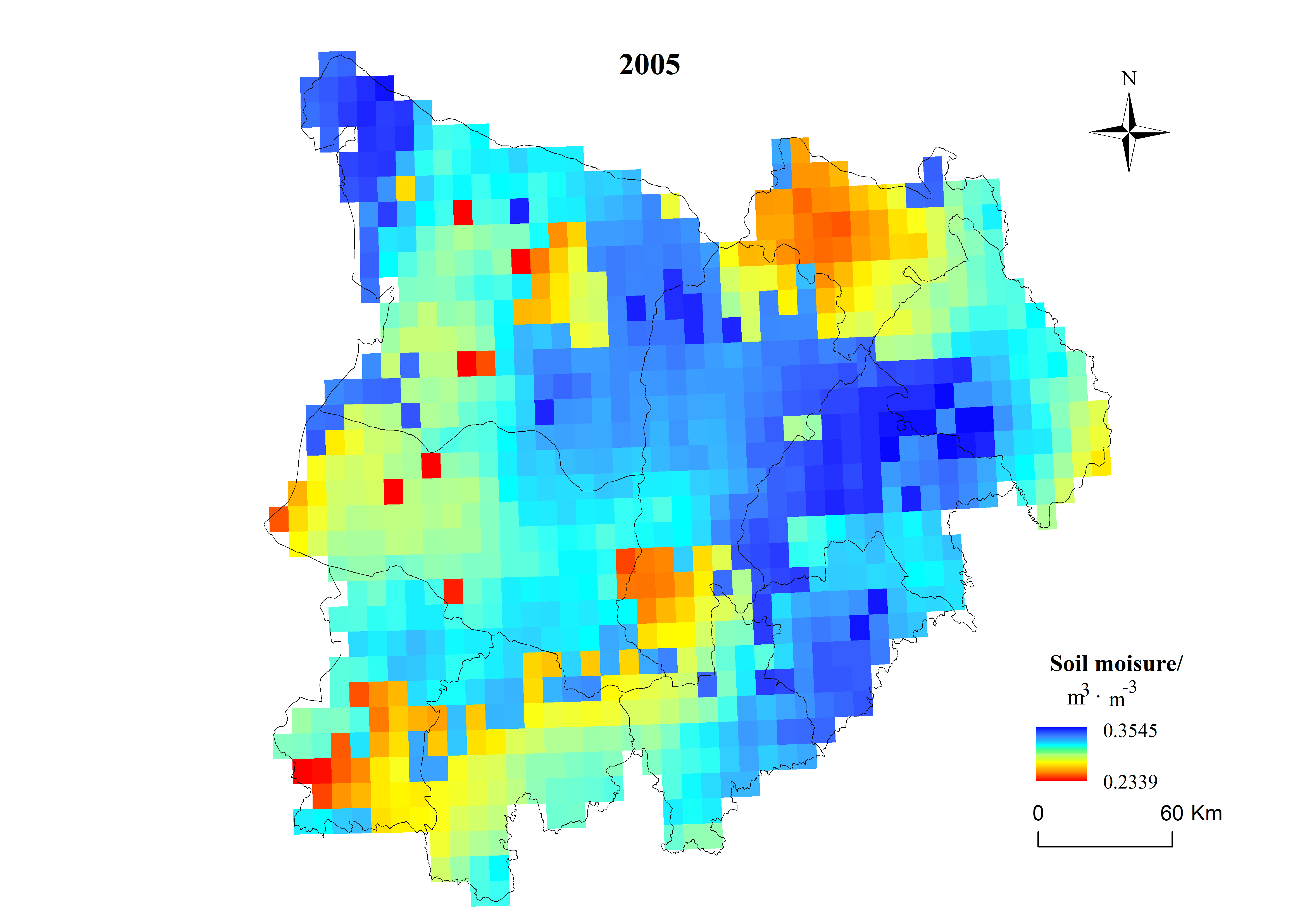

Supplement: S1 Fig — The average soil moisture at the underground depth of 0-10cm (a), 10-40cm(b), 40-100cm(c), and 100-200cm(d) in the study area in 2000–2020. (ZIP) [file pone.0292469.s001.zip › S1 Fig/(b)/2005.tif]

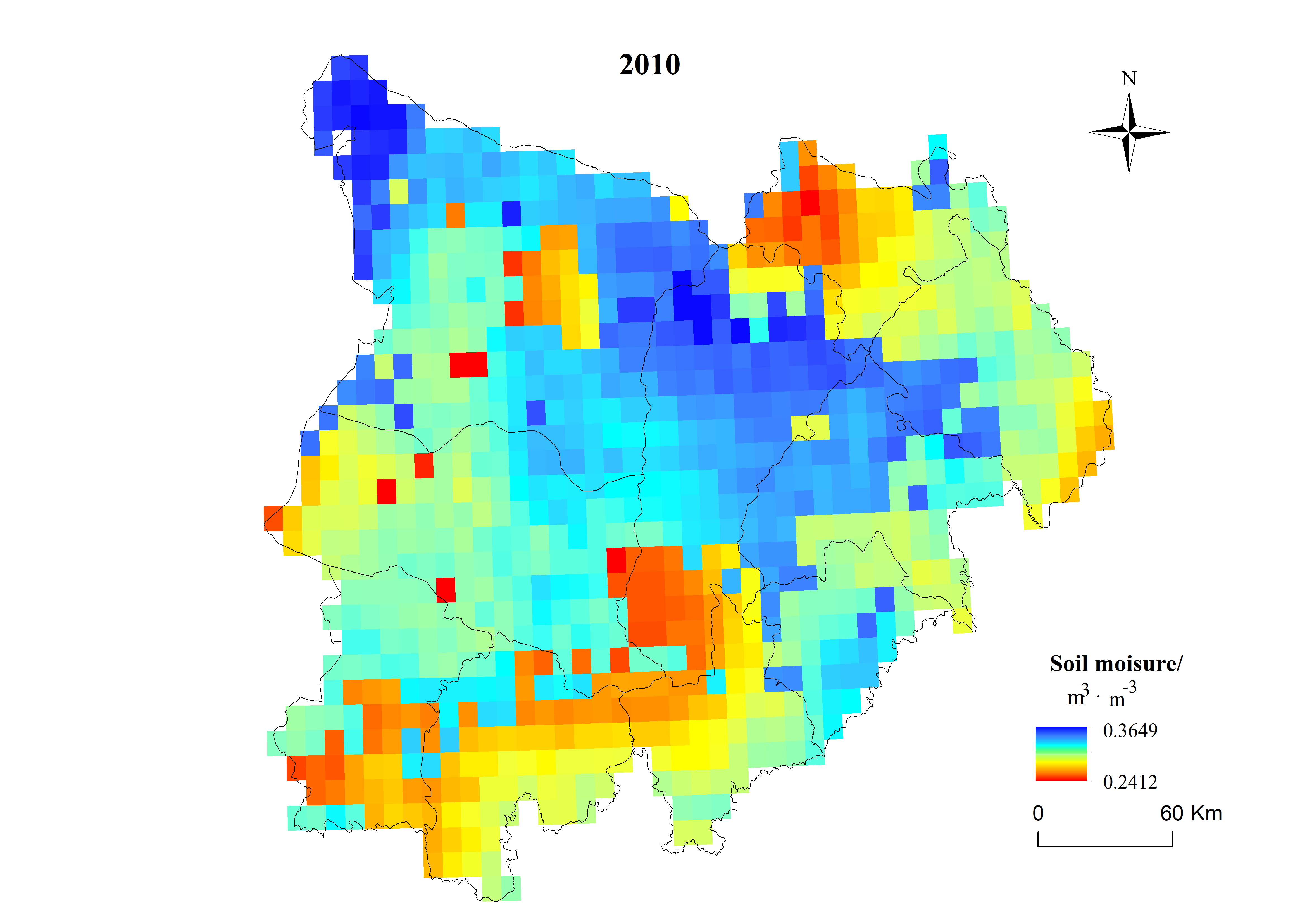

Supplement: S1 Fig — The average soil moisture at the underground depth of 0-10cm (a), 10-40cm(b), 40-100cm(c), and 100-200cm(d) in the study area in 2000–2020. (ZIP) [file pone.0292469.s001.zip › S1 Fig/(b)/2010.tif]

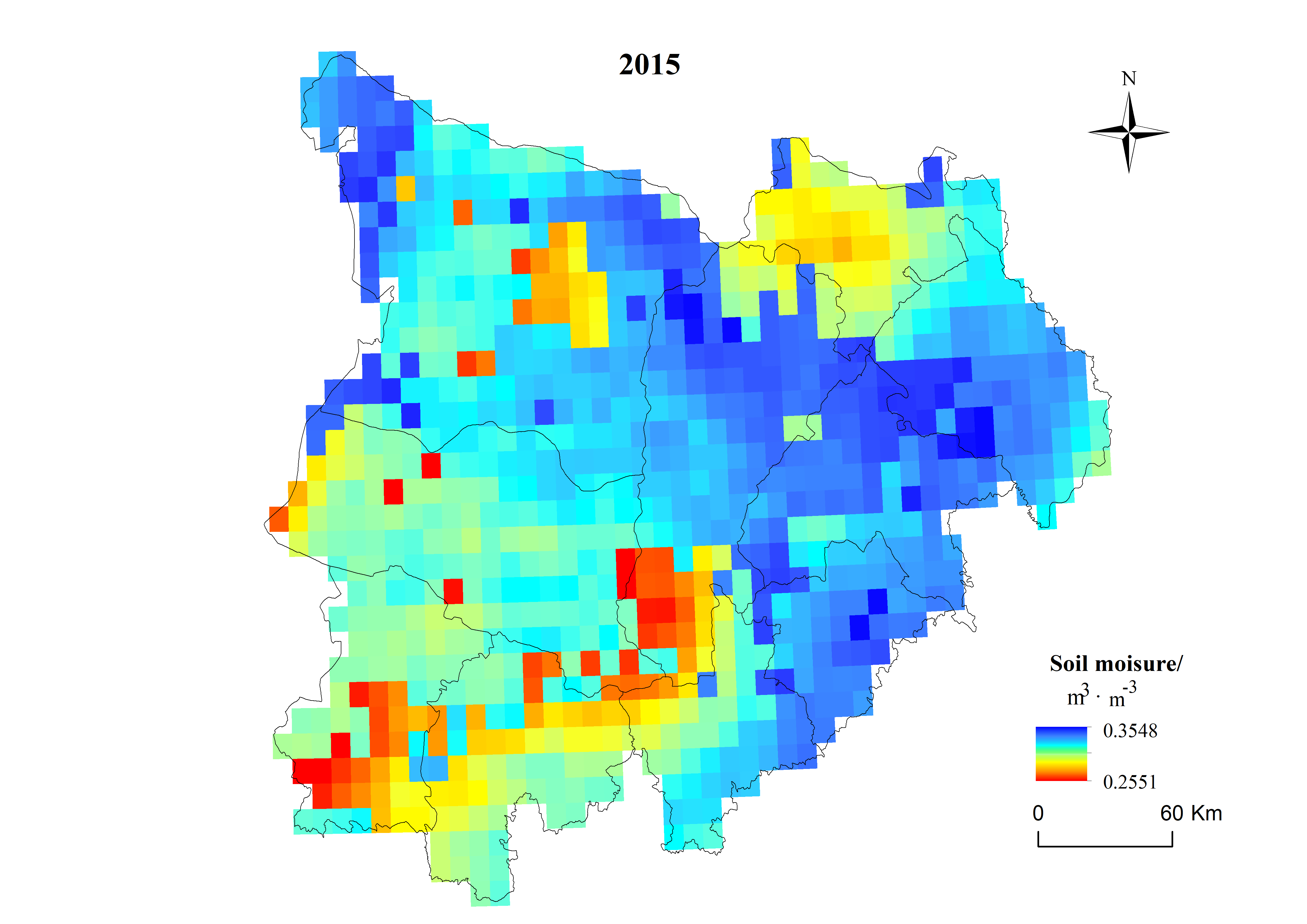

Supplement: S1 Fig — The average soil moisture at the underground depth of 0-10cm (a), 10-40cm(b), 40-100cm(c), and 100-200cm(d) in the study area in 2000–2020. (ZIP) [file pone.0292469.s001.zip › S1 Fig/(b)/2015.tif]

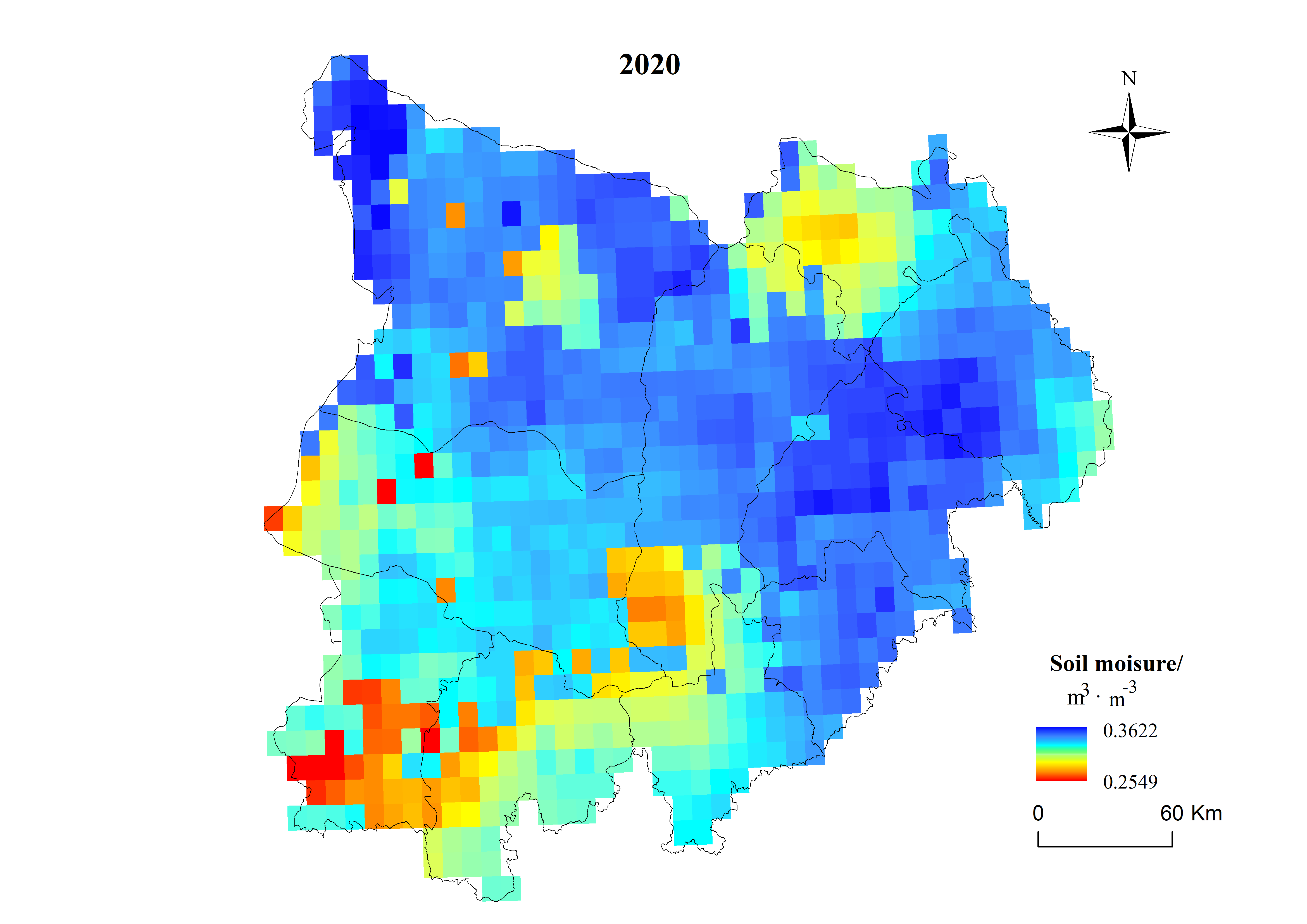

Supplement: S1 Fig — The average soil moisture at the underground depth of 0-10cm (a), 10-40cm(b), 40-100cm(c), and 100-200cm(d) in the study area in 2000–2020. (ZIP) [file pone.0292469.s001.zip › S1 Fig/(b)/2020.tif]

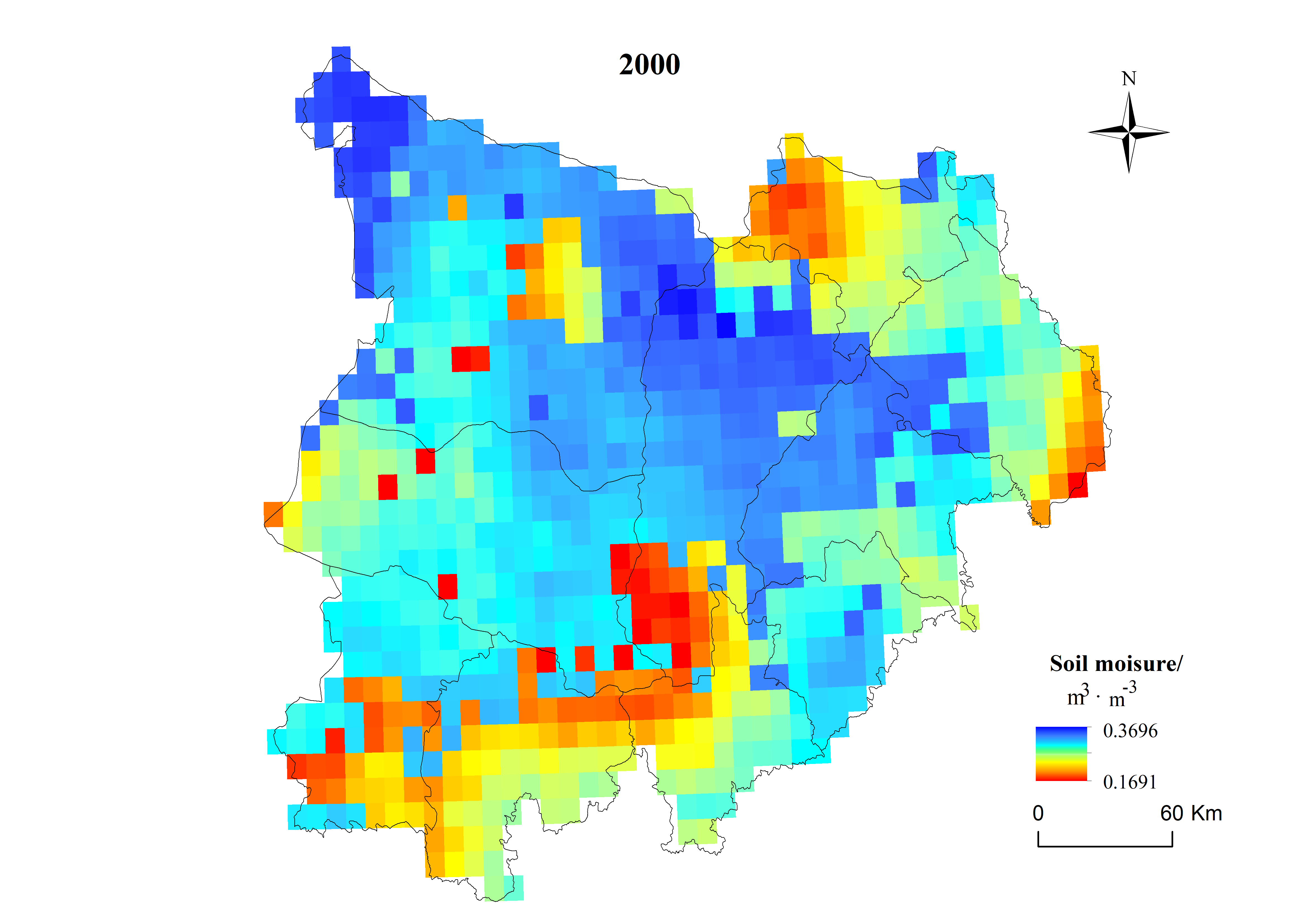

Supplement: S1 Fig — The average soil moisture at the underground depth of 0-10cm (a), 10-40cm(b), 40-100cm(c), and 100-200cm(d) in the study area in 2000–2020. (ZIP) [file pone.0292469.s001.zip › S1 Fig/(c)/2000.tif]

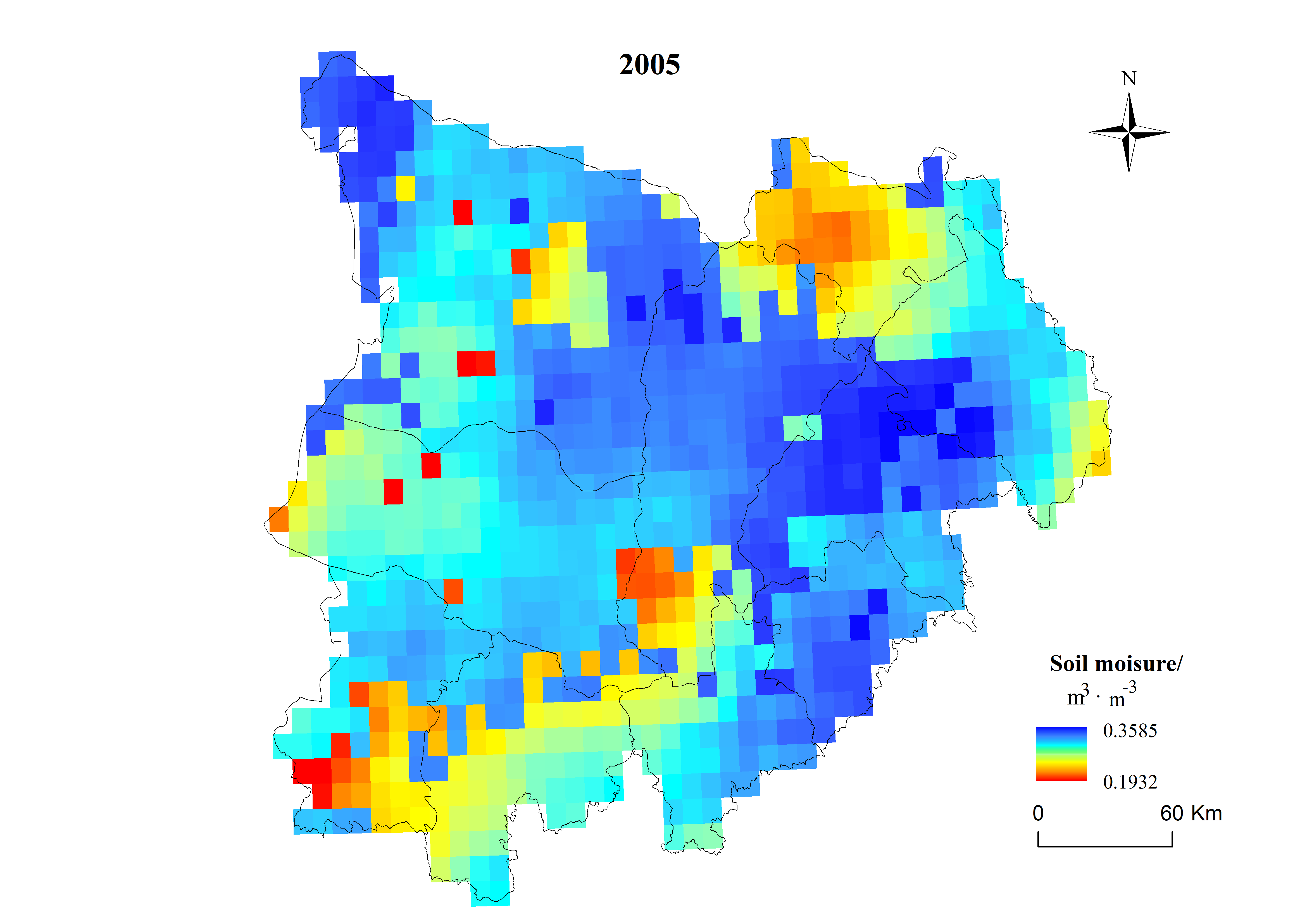

Supplement: S1 Fig — The average soil moisture at the underground depth of 0-10cm (a), 10-40cm(b), 40-100cm(c), and 100-200cm(d) in the study area in 2000–2020. (ZIP) [file pone.0292469.s001.zip › S1 Fig/(c)/2005.tif]

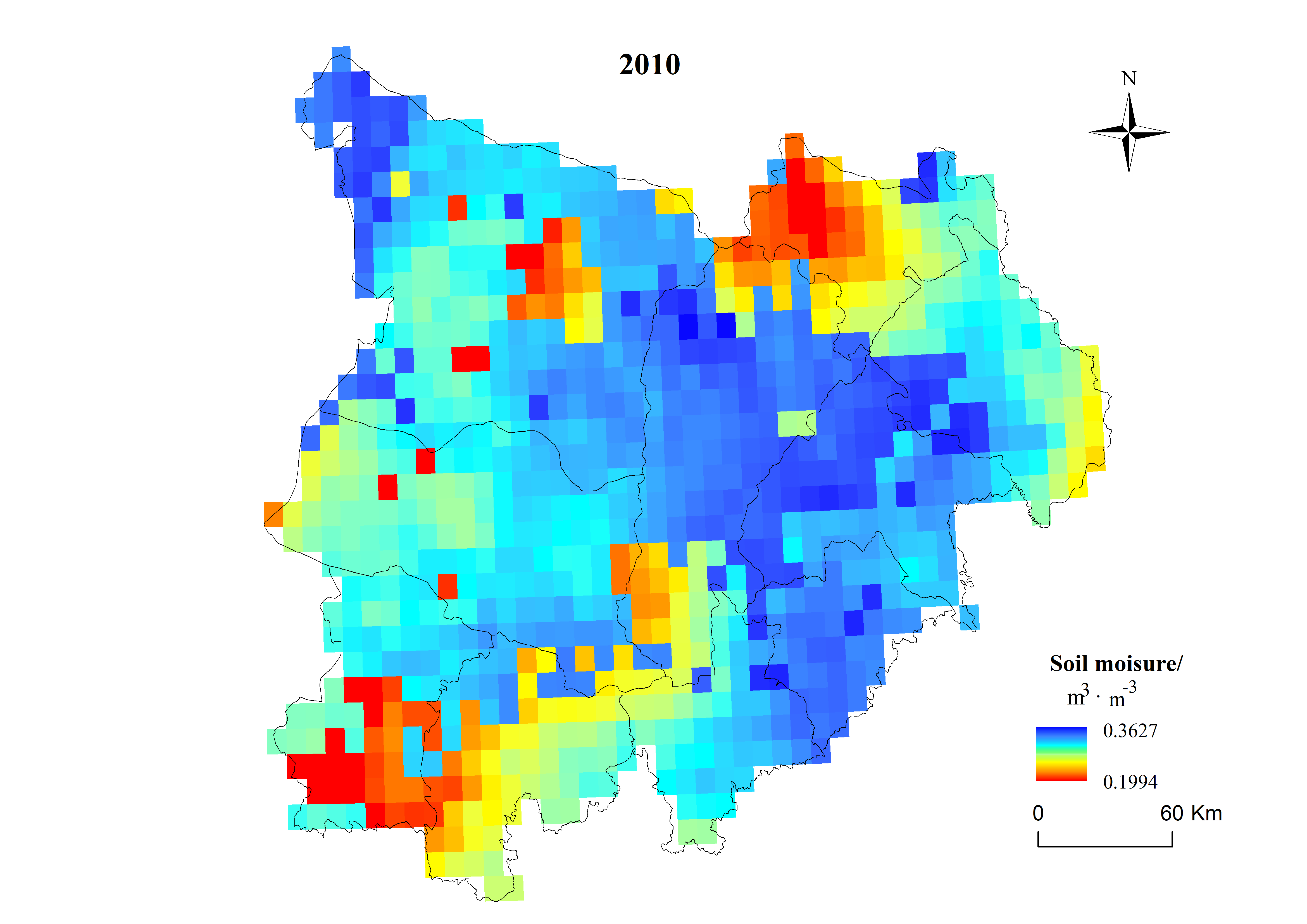

Supplement: S1 Fig — The average soil moisture at the underground depth of 0-10cm (a), 10-40cm(b), 40-100cm(c), and 100-200cm(d) in the study area in 2000–2020. (ZIP) [file pone.0292469.s001.zip › S1 Fig/(c)/2010.tif]

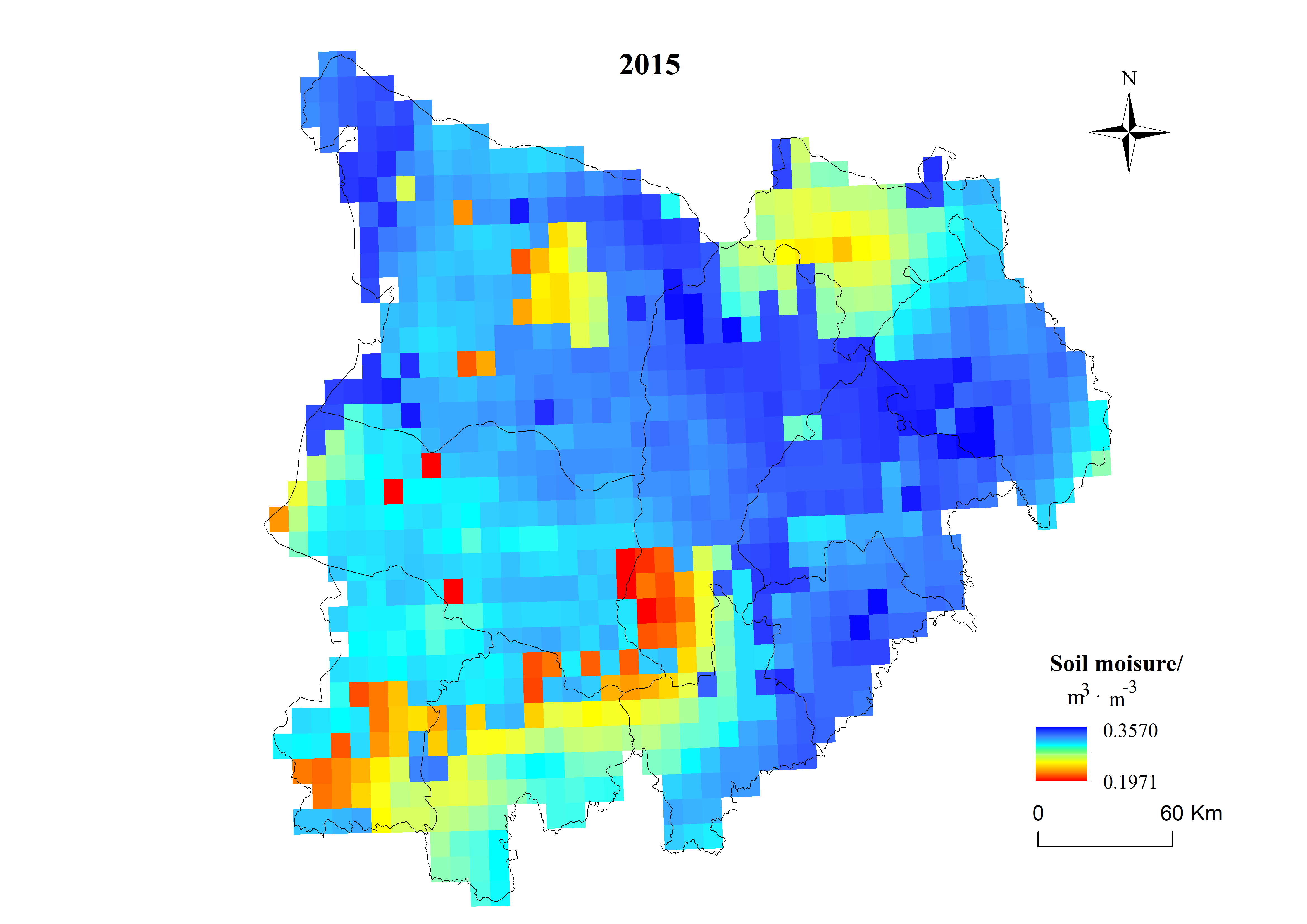

Supplement: S1 Fig — The average soil moisture at the underground depth of 0-10cm (a), 10-40cm(b), 40-100cm(c), and 100-200cm(d) in the study area in 2000–2020. (ZIP) [file pone.0292469.s001.zip › S1 Fig/(c)/2015.tif]

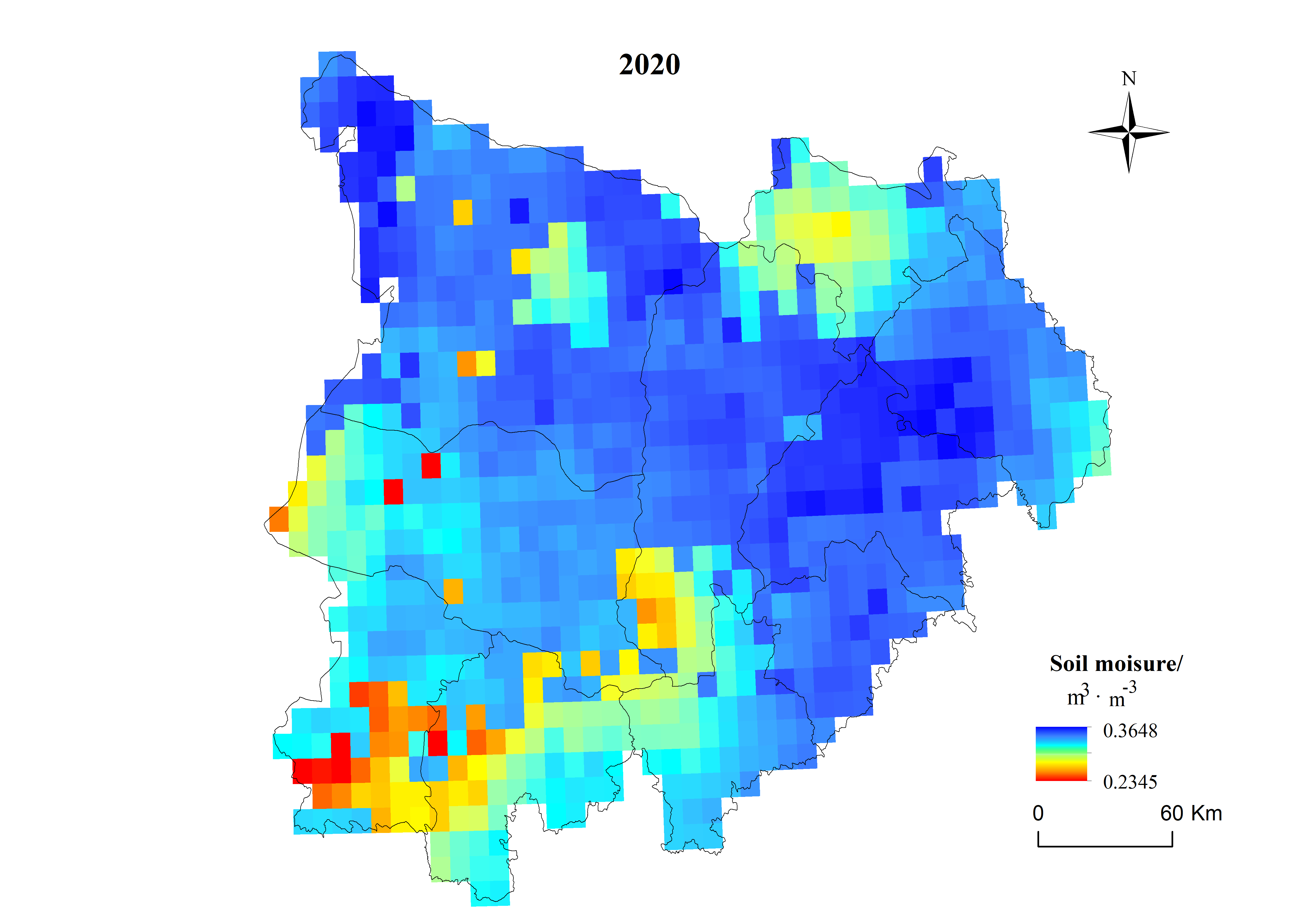

Supplement: S1 Fig — The average soil moisture at the underground depth of 0-10cm (a), 10-40cm(b), 40-100cm(c), and 100-200cm(d) in the study area in 2000–2020. (ZIP) [file pone.0292469.s001.zip › S1 Fig/(c)/2020.tif]

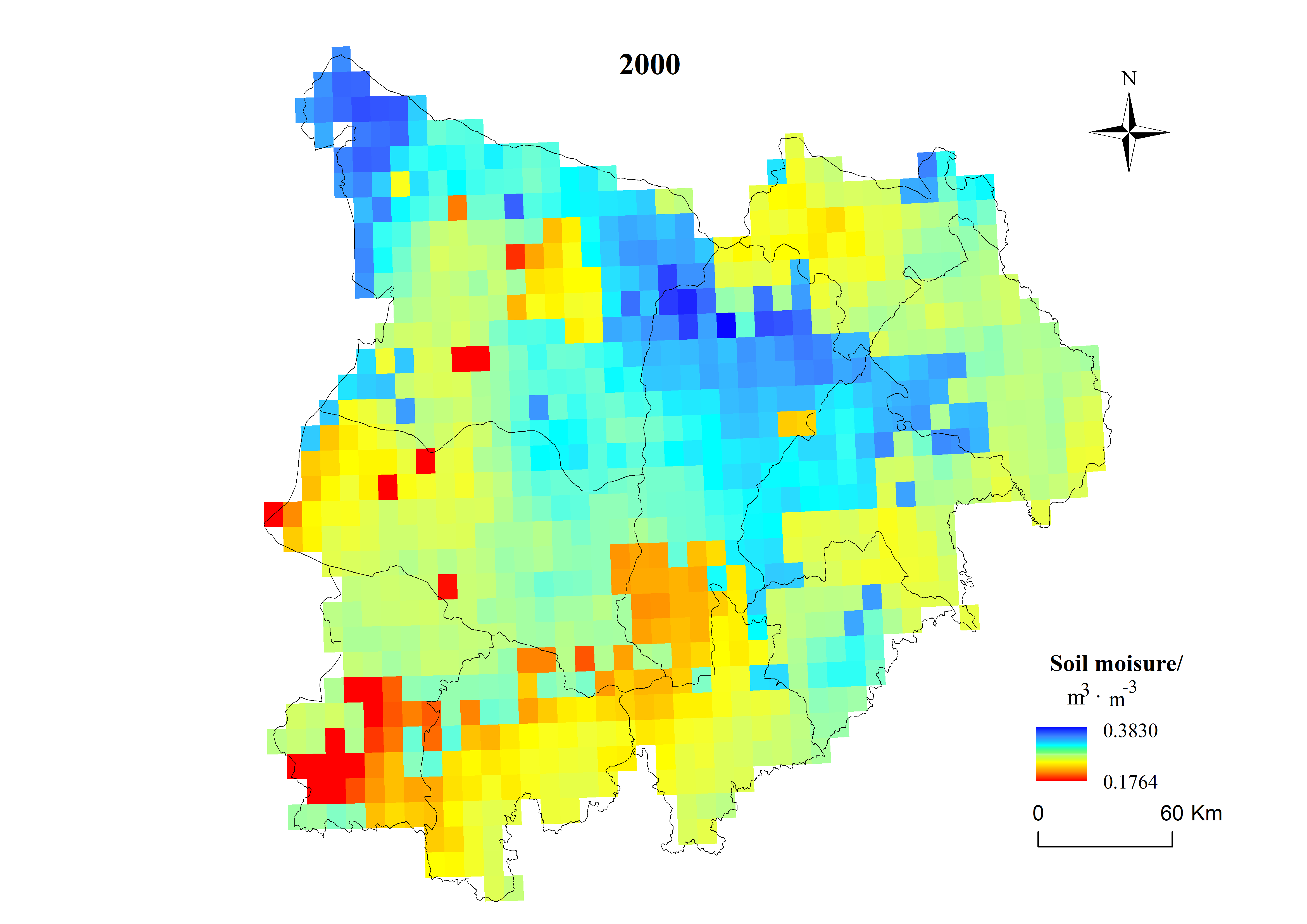

Supplement: S1 Fig — The average soil moisture at the underground depth of 0-10cm (a), 10-40cm(b), 40-100cm(c), and 100-200cm(d) in the study area in 2000–2020. (ZIP) [file pone.0292469.s001.zip › S1 Fig/(d)/2000.tif]

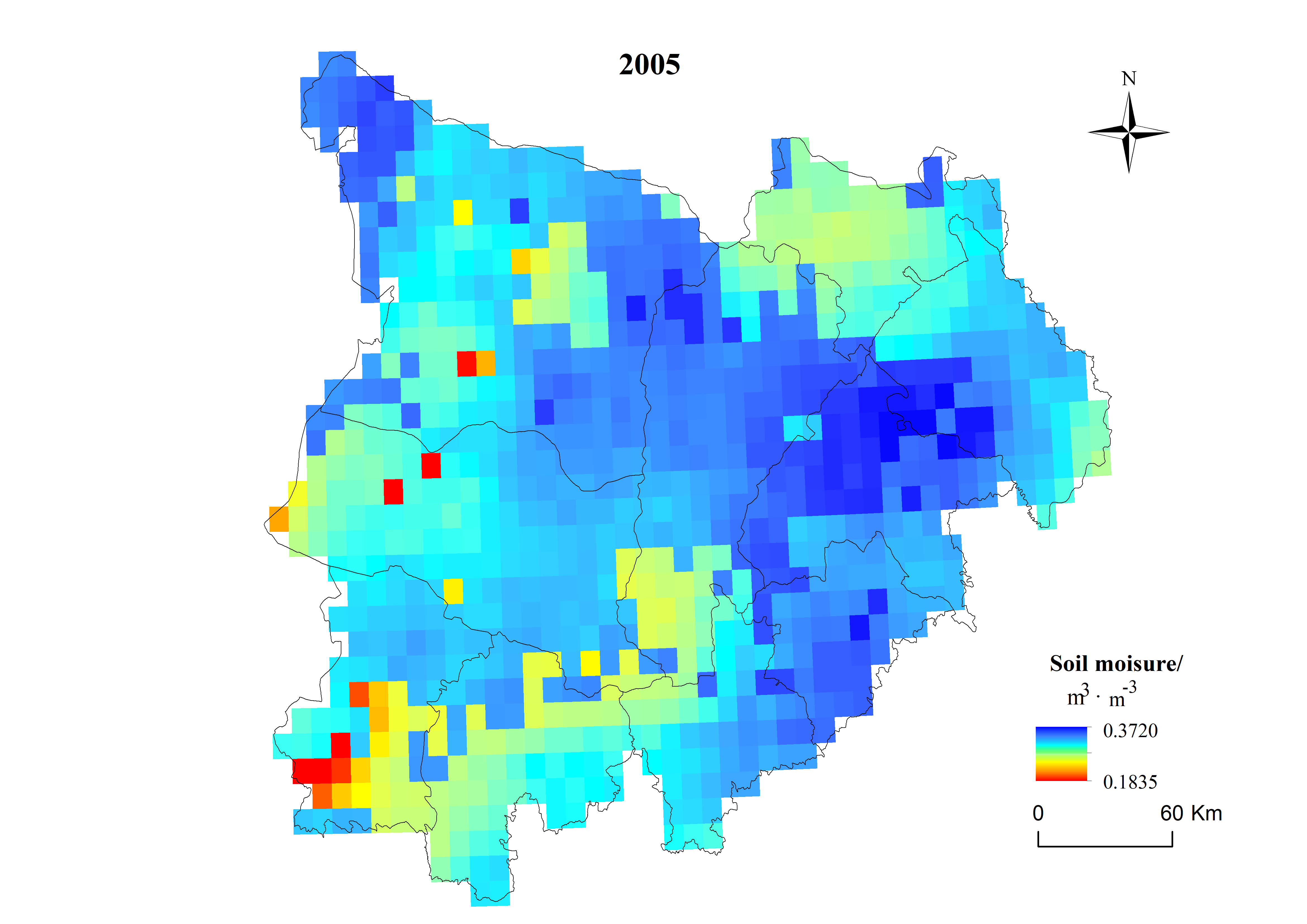

Supplement: S1 Fig — The average soil moisture at the underground depth of 0-10cm (a), 10-40cm(b), 40-100cm(c), and 100-200cm(d) in the study area in 2000–2020. (ZIP) [file pone.0292469.s001.zip › S1 Fig/(d)/2005.tif]

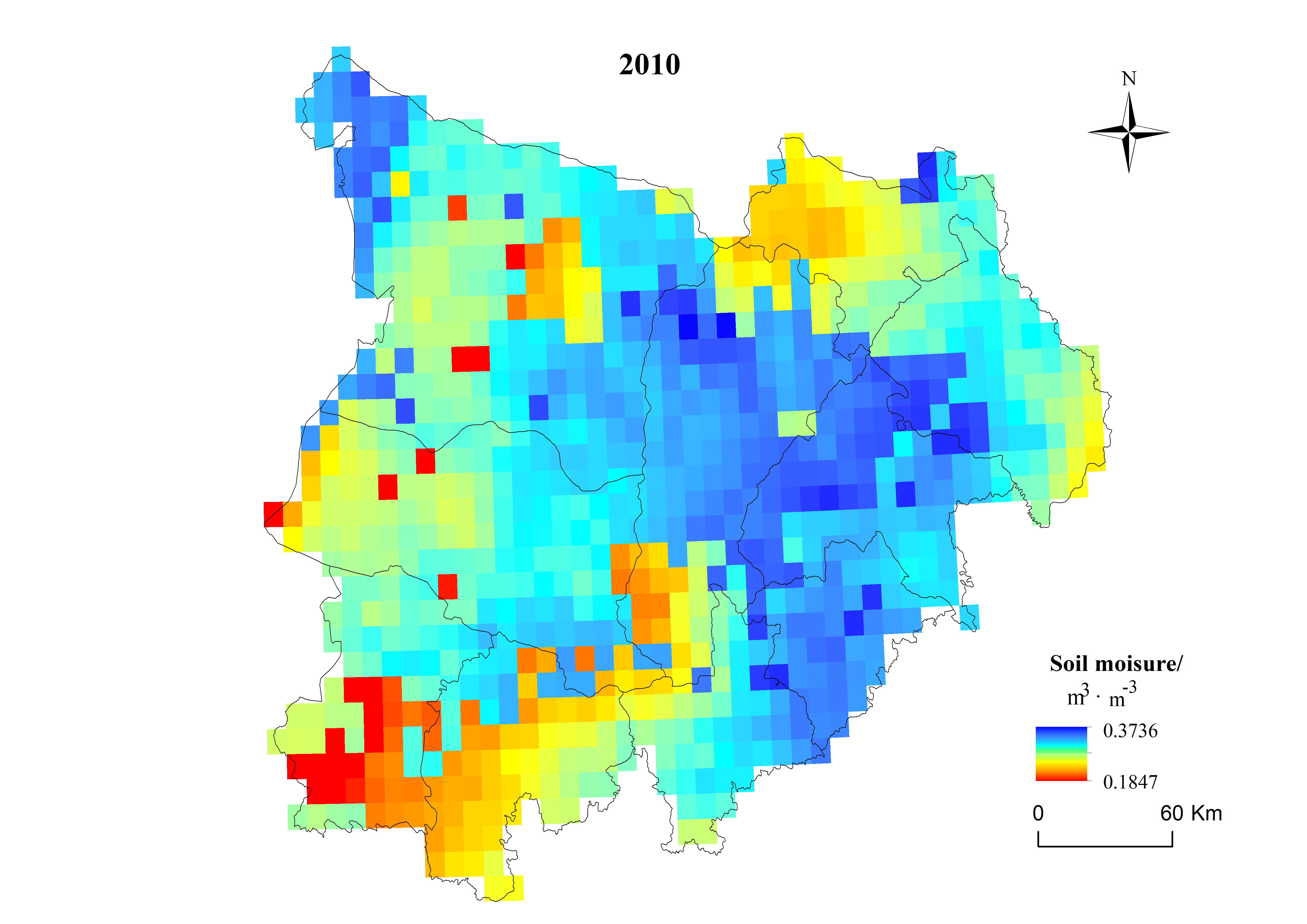

Supplement: S1 Fig — The average soil moisture at the underground depth of 0-10cm (a), 10-40cm(b), 40-100cm(c), and 100-200cm(d) in the study area in 2000–2020. (ZIP) [file pone.0292469.s001.zip › S1 Fig/(d)/2010.tif]

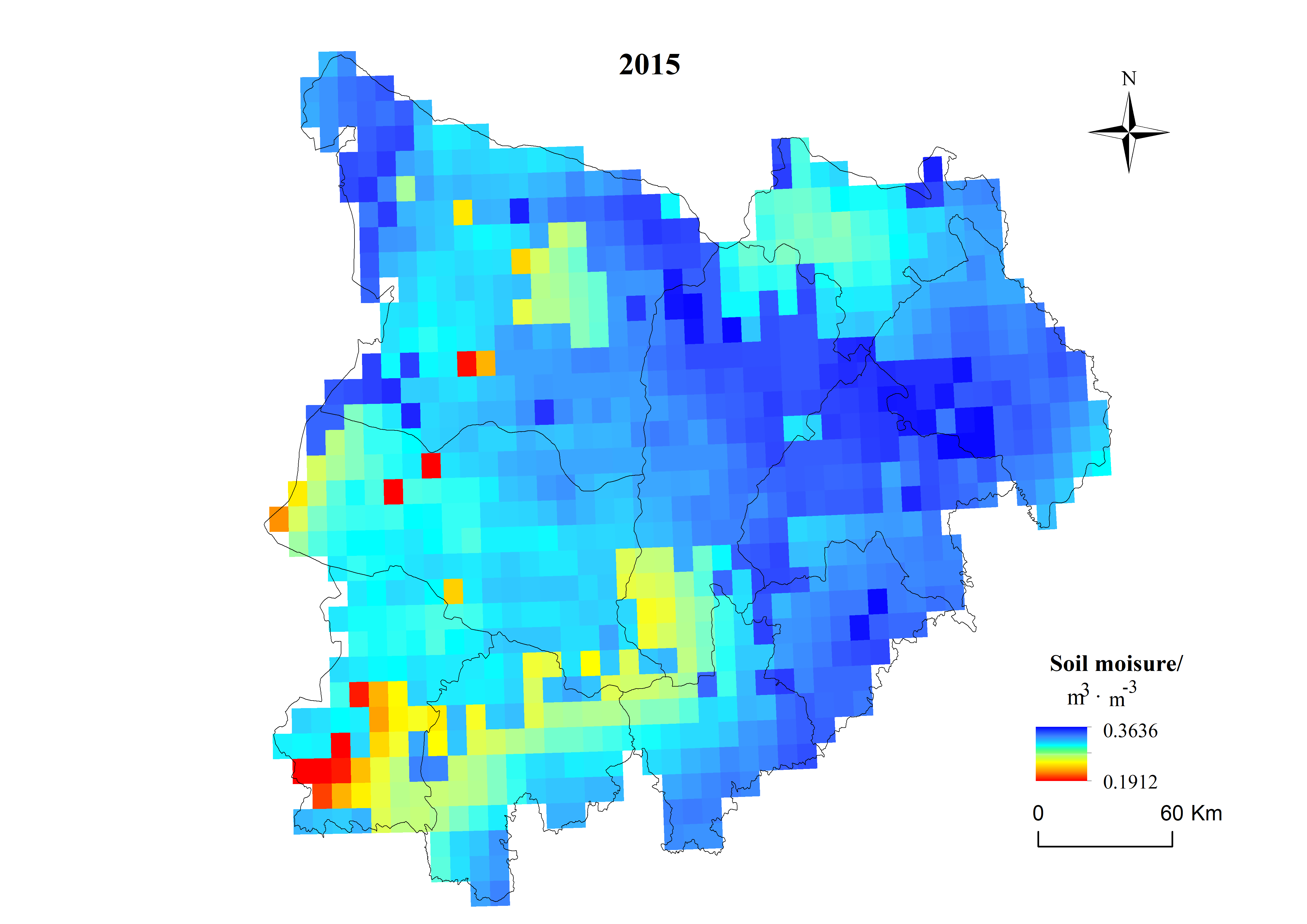

Supplement: S1 Fig — The average soil moisture at the underground depth of 0-10cm (a), 10-40cm(b), 40-100cm(c), and 100-200cm(d) in the study area in 2000–2020. (ZIP) [file pone.0292469.s001.zip › S1 Fig/(d)/2015.tif]

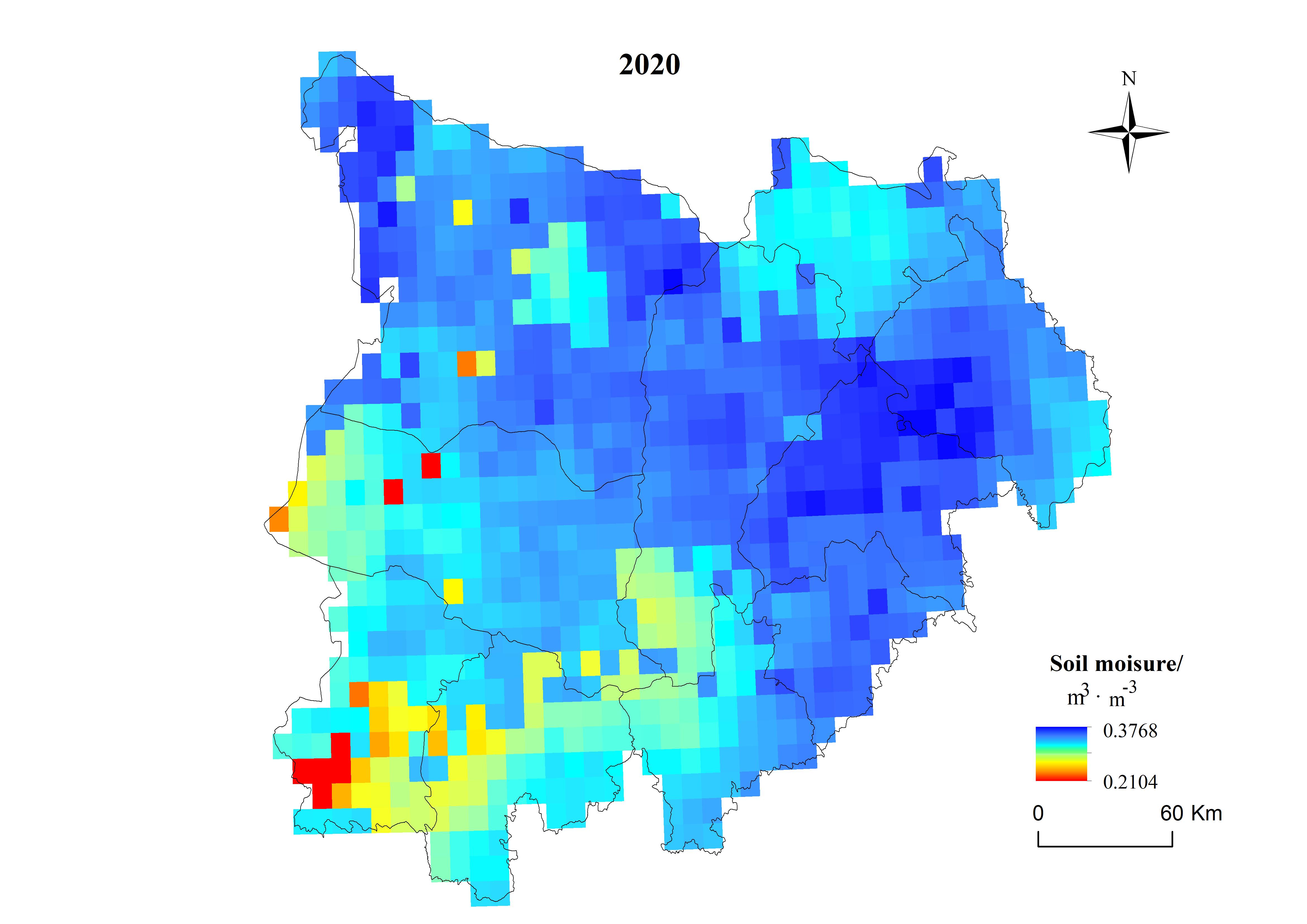

Supplement: S1 Fig — The average soil moisture at the underground depth of 0-10cm (a), 10-40cm(b), 40-100cm(c), and 100-200cm(d) in the study area in 2000–2020. (ZIP) [file pone.0292469.s001.zip › S1 Fig/(d)/2020.tif]

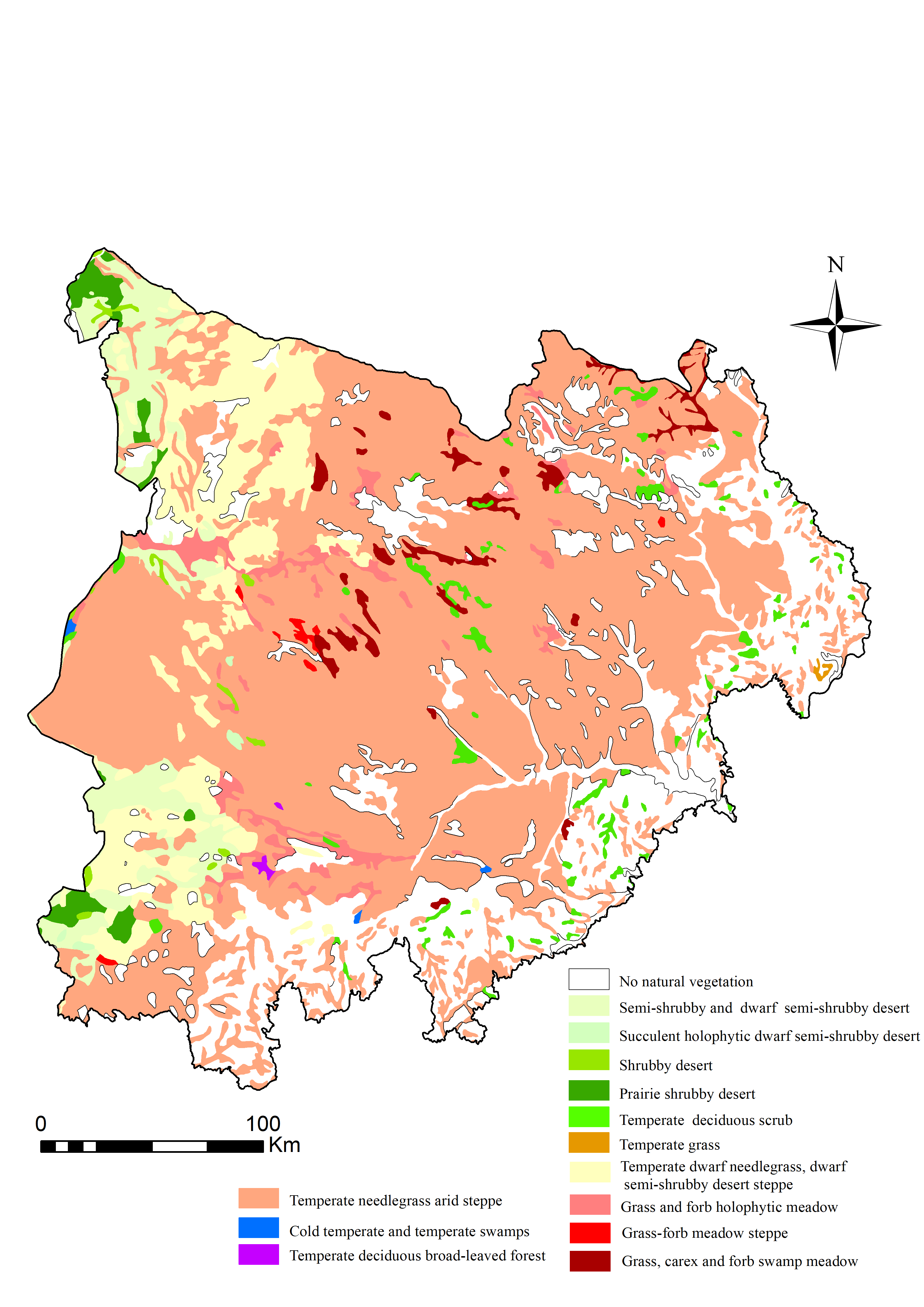

Supplement: S2 Fig — (TIF) [file pone.0292469.s002.tif]

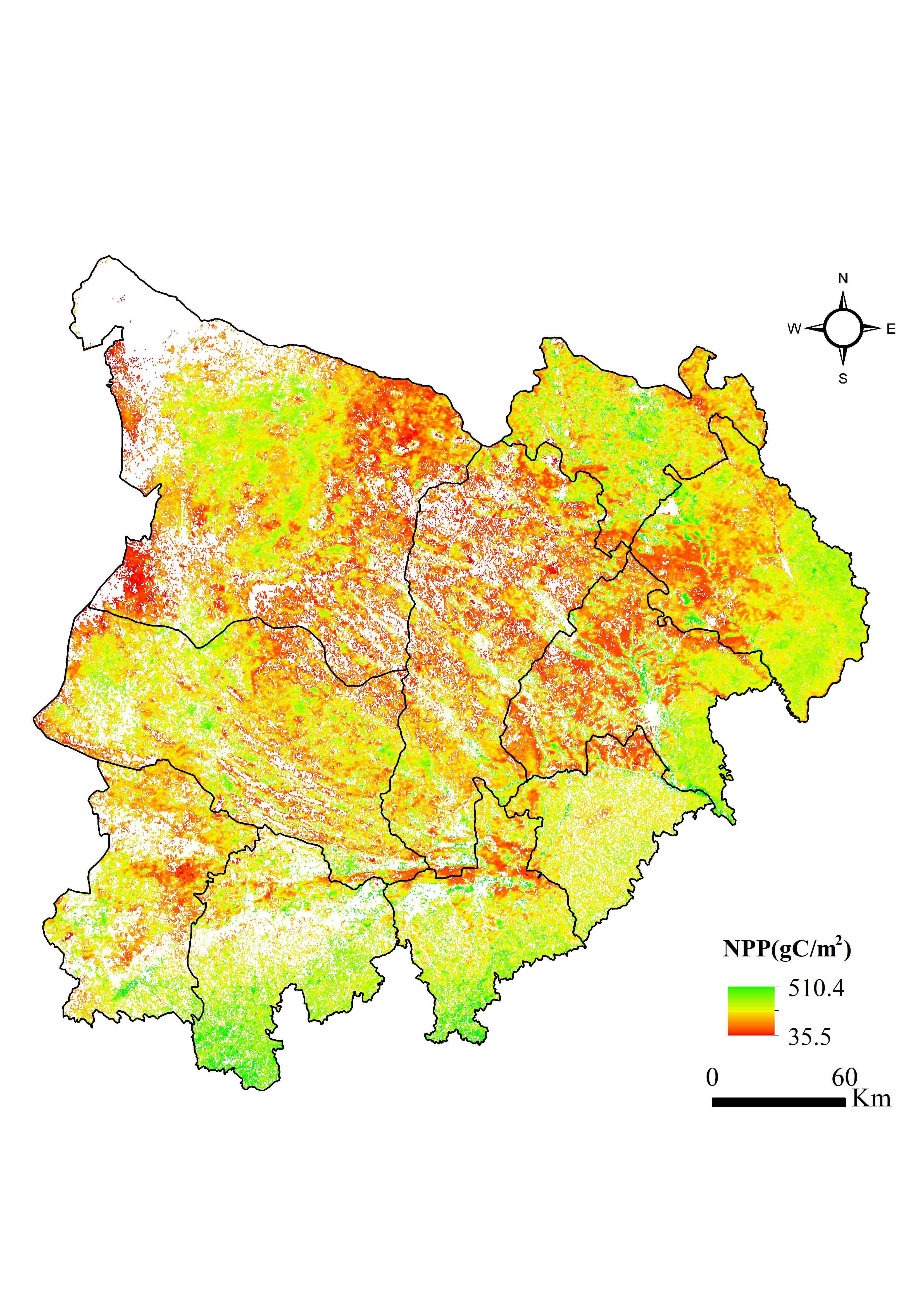

Supplement: S3 Fig — (ZIP) [file pone.0292469.s003.zip › S3 Fig/NPP(right).tif]

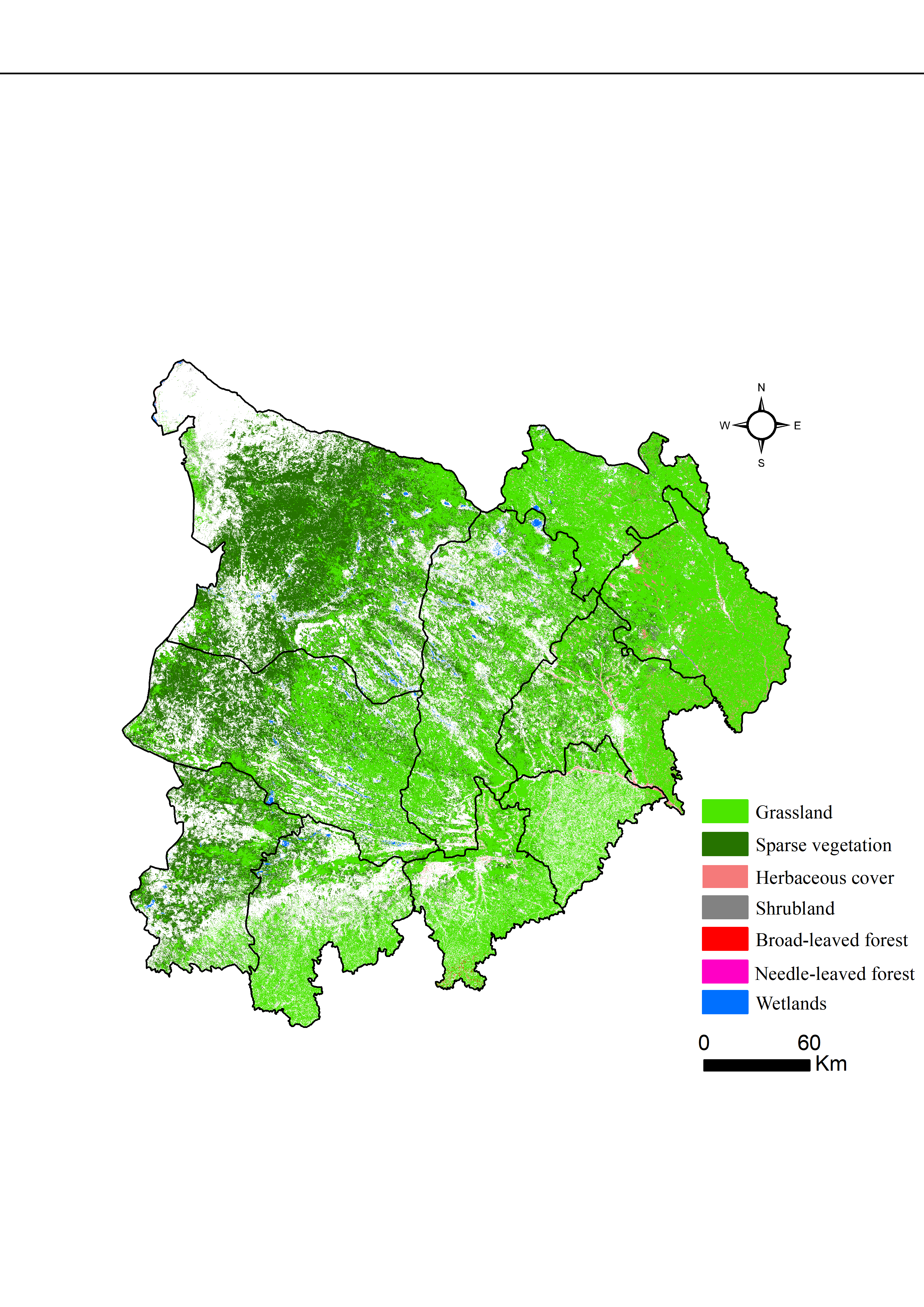

Supplement: S3 Fig — (ZIP) [file pone.0292469.s003.zip › S3 Fig/Vegetation types(left).tif]

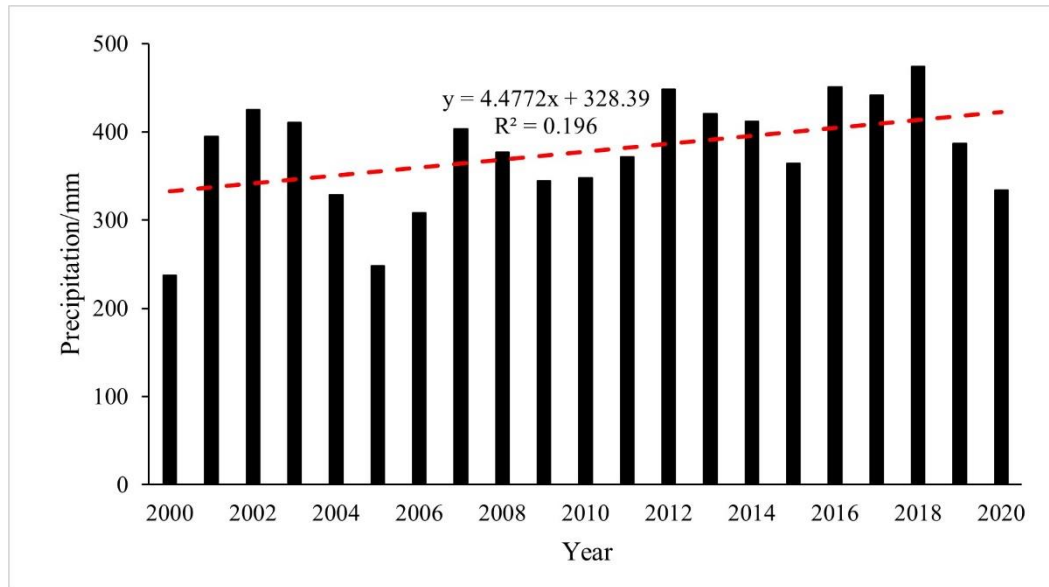

Supplement: S4 Fig — (PDF) [file pone.0292469.s004.pdf]

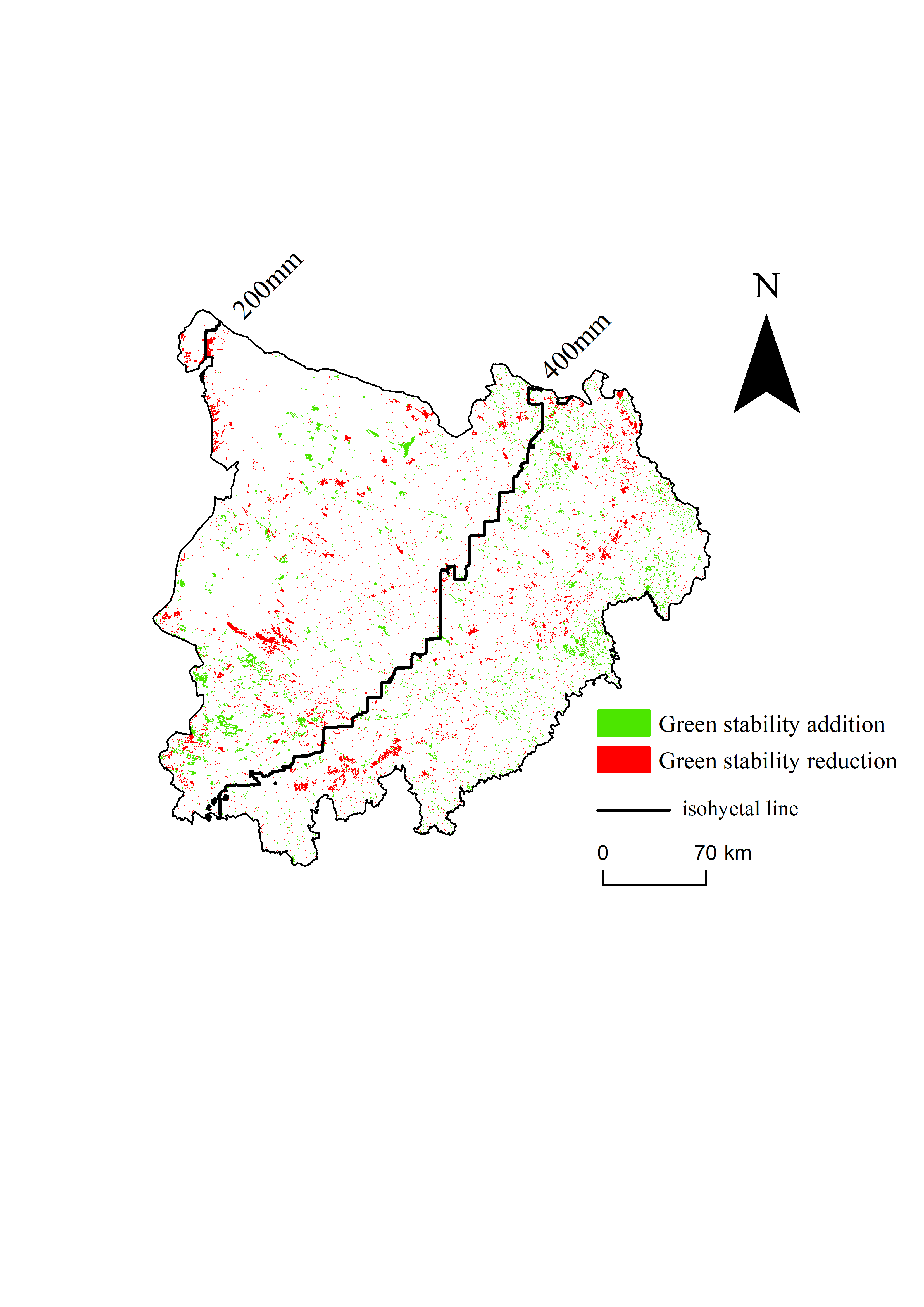

Supplement: S5 Fig — (TIF) [file pone.0292469.s005.tif]
